# WTAP and BIRC3 are involved in the posttranscriptional mechanisms that impact on the expression and activity of the human lactonase PON2

**DOI:** 10.1038/s41419-020-2504-2

**Published:** 2020-05-07

**Authors:** Teresa Maria Carusone, Giovanna Cardiero, Mariangela Cerreta, Luigi Mandrich, Oscar Moran, Elena Porzio, Giuliana Catara, Giuseppina Lacerra, Giuseppe Manco

**Affiliations:** 1 Institute of Biochemistry and Cell Biology (IBBC, CNR), National Research Council, Naples, Italy; 2 Institute of Genetics and Biophysics “Adriano Buzzati Traverso”, (IGB-ABT, CNR), National Research Council, Naples, Italy; 3 Institute of Biophysics (IBF, CNR), National Research Council, Genoa, Italy

**Keywords:** Ubiquitylation, Hydrolases, SAXS

## Abstract

The activity of human paraoxonase 2 (PON2) is rapidly reduced in cells incubated with the bacterial quorormone 3-Oxo-dodecanoyl Homoserine Lactone (3OC12HSL), an observation that led to hypothesize a fast PON2 post-translational modification (PTM). Recently, we detected a 3OC12HSL-induced PTM in a cell-free system in which a crude extract from 3OC12HSL-treated HeLa cells was able to inactivate and ubiquitinate at position 144 a recombinant PON2. Here we show the occurrence of this and new PTMs on PON2 in HeLa cells. PTMs were found to gather nearby the two SNPs, A148G, and S311C, that are related to type-2 diabetes and its complications. Furthermore, we detected a PTM nearby a 12 amino acids region that is deleted in PON2 Isoform 2. An in vitro mutation analysis showed that the SNPs and the deletion are involved in PON2 activity and suggested a role of PTMs on its modulation, while a SAXS analysis pointed to Isoform 2 as being largely unstructured, compared to the wild type. Besides, we discovered a control of PON2 expression *via* a putative mRNA operon involving the Wilms tumor 1 associated protein (WTAP) and the E3 ubiquitin ligase (E3UbL) baculoviral IAP repeat-containing 3 (BIRC3).

## Introduction

The human paraoxonase (PON) family comprises three highly conserved lactonases: PON1, PON2, and PON3^1,2^. PON1 and PON3 are predominantly secreted from the liver into the blood; PON2 instead is an intracellular protein found almost in every tissue. In particular PON2 has been found at the perinuclear region, in the endoplasmic reticulum (ER), in the mitochondria and on the external face of the plasma membrane^3–5^. Among the three PONs, PON2 shows the highest lactonase activity^6,7^. On account of its localization on the plasma membrane and activity, PON2 is considered to be the first line of defence against Gram-negative bacteria infections^7^. The bacterial signal 3-Oxo-dodecanoyl Homoserine Lactone (3OC12HSL) is the major player of quorum sensing (QS) in Gram-negative bacteria^8^. It has also been shown to enter into the eukaryotic cells, triggering apoptosis in many cell types (including cancer cells)^9^, and modulating the host immune responses, hence the term quorormone^10^. Due to its lactonase activity PON2 controls the bacterial QS process, regulating the biofilm formation and the production of virulence factors^7^. In some epithelial cells, PON2 serves also a pro-apoptotic function by degrading 3OC12HSL^9^. However, several in vitro and in vivo studies indicate that PON2 in addition to the lactonase activity displays also an anti-ROS activity^3,11–15^. Because it is well established that oxidative stress and chronic inflammation are closely linked to cell death^16^ this provides a mechanistic direction for epidemiological studies that show a link between PON2 and numerous inflammatory diseases, such as type 2 diabetes and cancer^17^ (see Supplementary information).

In cells, 3OC12HSL can quickly inactivate PON2 lactonase activity^14^. In a cell-free system made of a rPON2 incubated with HeLa crude extracts, we reported that it is possible to recapitulate and study 3OC12HSL-mediated PON2 inactivation. Enzyme inactivation was paralleled by ubiquitination at position K144^18^. In this paper, we proceed further on this lane by approaching the study of the post-transcriptional regulation of PON2 activity and expression.

RNA-binding proteins (RBPs) have been shown to control the expression of many genes by binding to the respective mRNA species, encoding proto-oncogenes, growth factors, cytokines, transcription factors, and other proteins in various cell types^19^. The 3′ untranslated (3′ UTRs) and the 5′ UTRs are the transcript target sequences involved in the RBPs binding and mediating the formation of the ‘RNA operon’. This is a functional unit in which multiple physiologically related transcripts can be co-ordinately regulated during splicing, export, stability, localization, and translation. These subpopulations of mRNAs bind the same RBPs in a dynamic manner because each mRNA can join different RNA operons^20–23^. Some of us recently reported an example of RNA operon for human major histocompatibility complex II gene^24,25^.

Based on the analysis of the regulatory function of the 3′ UTR sequences^21^, we identified a cluster of 21 human genes comprising PON2 characterized by the sharing at the 3′ UTR leader of the conserved sequence: UUUCCUUAAAAU^21,26^ (Supplementary Table 6). We hypothesized a post-transcriptional control of PON2 via an mRNA operon relying upon RBPs recognizing the conserved sequence. Interestingly, among the 21 found genes, two were newly identified RBPs namely: the Wilms tumor 1-associated protein (WTAP)^27–29^ and the tryptophan and aspartate repeat domain 36 (WDR36)^30,31^. WTAP has been reported to bind the 3′ UTR of CCNA2 (cyclin A2), which enhances its stability thereby regulating G2/M cell-cycle transition^28^, and to induce cancer, if deregulated, such for example through the stabilization of Fak mRNA in pancreatic cancer^29^. Recently, the role of WDR36 as RBP has emerged from a global approach of RBPs identification in HeLa cells^31^ and its involvement in the glaucoma disease^30^. Interestingly, 4 out of the 21 members of the cluster encode E3 ubiquitin ligases (E3UbL) or elements of complexes endowed with E3UbL activity (see references of Supplementary Table 5). Hypothesizing a co-posttranscriptional regulation mechanism including the PON2 gene and modifiers, we explored this concept in HeLa and in A549 cells lines. Furthermore, we detected not yet confirmed PON2 isoforms and post-translational modifications, likely affecting activity.

## Material and methods

### Cell cultures

Human cervical HeLa (ATCC) and human lung cancer A549 (ATCC) cell lines free of mycoplasma contamination were maintained, respectively, in RPMI medium 1640 (Gibco by Life Technologies, Paisley, Scotland), and DMEM medium (Lonza, Verviers, Belgium) with l-glutamine, supplemented with 10% fetal bovine serum (Gibco by Life Technologies), 100 U/ml penicillin/streptomycin (Microgem Laboratory Research, Pozzuoli, Italy) at 37 °C in 5% CO_2_.

Experiments were mostly performed on HeLa cells except when indicated.

### 3OC12HSL treatment of HeLa cells

2.5 × 10^5^ HeLa cells were seeded in 35 mm disc plates in 2 ml of complete medium and incubated at 37 °C in 5% CO_2_. Twenty-four hours later (at ~70% confluence) the cell were treated with DMSO or 3OC12HSL (100 μM) in DMSO. The plates, two for each point, were incubated for the given time. At the times reported in Fig. 4, the cells were rinsed with cold phosphate-buffered saline (PBS)-EDTA and one plate for each point was lysed with tri-reagent, for the mRNA analysis, and the other one with the lysis buffer for the protein analysis.

### Transfection with siRNAs

The cell lines were transfected with siGENOME SMARTPool siRNAs using DarmaFECT 1 as transfection reagent (Dharmacon, GE Healthcare, USA), according to the manufacturers’ instructions. Twelve hours before transfection, 2–4 × 10^4^ HeLa or A549 cell lines were seeded in 500 μl of growth medium in 24 wells cell culture plates or 1.5 × 10^5^ cells in 2 ml of medium in six wells cell culture plates. In separate tubes the siRNA, at the desired concentration, and 1.33 μl (24 wells plate) or 6.66 μl (six-well plate) of DarmaFECT 1 were diluted in 50 μl (24 well) or 200 μl (six well) of serum-free medium and incubated for 5 min at room temperature. Complexes were formed by combining the two diluted components, mixing by pipetting carefully and incubating for 20 min at room temperature. Subsequently, the medium from the well was removed, and 0.400 ml (24 well) or 1.6 ml (six well) of antibiotic-free complete medium was added to the complex, mixed and pipetted in each well, containing cells at 50–70% of confluence. Each experiment was carried out in triplicate or duplicate wells and the complexes were prepared multiplying the volumes. The plates were incubated for 24–48 h at 37 °C in 5% CO_2_. Elapsed the incubation time, the transfected cells were washed with PBS and harvested in TRI Reagent for RNA analysis or in PBS/EDTA and shared for both mRNA and protein analysis. Total RNA was isolated with TRI Reagents (Molecular Research Center, Cincinnati, OH, USA) according to the manufacturer’s instructions. The quality and quantity of the resulting RNA were determined by gel electrophoresis and NanoDrop ND-1000 spectrophotometer (Thermo Fisher Scientific, USA).

### Dose curve transfections

Dose curve transfections to identify the optimum siRNAs concentration for the silencing of our target genes were performed. Three different concentrations of siRNAs (12.5, 25, and 50 nM), were transfected. Each experiment included the siGENOME SMARTPool siRNAs for: the target genes WTAP #M-017323-02-0005, WDR36 #M-017004-01-0005, and BIRC3 #M-004099-02-0005, GAPDH #D-001140-01-05 (as endogenous targeting) and the non-targeting siGENOME pool #D-001206-14-05 (as negative control). Twenty-four hours later, cells were washed with PBS and harvested in TRI Reagent (Molecular Research Center, Cincinnati, OH, USA).

### Reverse transcription PCR and quantitative real-time PCR

One microgram of total RNA was treated with RNase-free DNase I amplification grade (Invitrogen, USA) and retro-transcribed with random primers using Superscript IV (Invitrogen, USA). The quantitative real-time PCR was performed with the use of CFX connect real-time PCR detection system (Bio-Rad, Hercules, CA, USA) and the SsoAdvanced Universal SYBR Green Supermix (Bio-Rad), following the manufacturer’s instruction. The primers reported in Supplementary Tables 1 and 7, synthesized by Eurofins, were designed using the “Primer Express” software v.3.0 (Applied Biosystems, Warrington, UK) and a Primer-BLAST program available at http://www.ncbi.nlm.nih.gov/tools/primer-blast/^32^. All samples were analyzed in duplicate, using a 30 ng of cDNA. ACTB was used as the reference gene. Relative expression of genes was calculated by the 2[−∆∆*C*(*T*)] method.

### Cell extracts

HeLa and A549 cells (2 × 10^7^) were scraped into PBS and pelleted. Pellet was suspended in 0.2 ml of lysis buffer (50 mM Tris/HCl pH 8.5, 150 mM NaCl, 1% Triton or Nonidet P40) supplemented with 8 μl of a 25× complete protease inhibitor cocktail (Roche, Monze, Italy). The cell lysate was maintained at constant agitation at 4 °C for 30 min and then centrifuged at 8000 × *g* in a microcentrifuge at 4 °C for 20 min. An equal amount of total proteins from the total lysate, soluble fraction and pellet were analyzed by 12.5% SDS–PAGE and western blot.

### Western blotting and immunodetection

Protein samples were fractionated on 12% SDS–PAGE and electroblotted onto Porablot nitrocellulose (NC) membranes (Macherey-Nagel, Düren, Germany) using a semidry transfer apparatus (Bio-Rad). Membranes were blocked with Tris-buffered saline, 0.05% Tween 20, and 5% nonfat dried milk for 1 h; washed with Tris-buffered saline containing Tween 20 (0.05% v/v), and then incubated overnight at 4 °C with specific primary antibodies.

After washing, the membranes were incubated for 1 h with horseradish peroxidase-conjugated secondary antibodies. Specific bands were detected using Luminata Crescendo Western HRP Substrate (Millipore, Milan, Italy) following the manufacturer’s suggested protocol. Densitometry was performed with the program ImageJ available free of charge at imagej.nih.gov/ij/download/.

The antibodies used for Western Blotting and Immunoprecipitation (IP) were the following: mouse-anti-glycerin-aldehyde 3-phosphate-dehydrogenase (GAPDH-6C5); mouse monoclonal anti-PON2 (C-5, sc-374158 from Santa Cruz Biotechnology, Heidelberg, Germany), rabbit polyclonal anti-PON2 serum produced by Covalab (Villeurbanne, France); mouse monoclonal anti-Ubiquitin (P4D1) from Santa Cruz Biotechnology, (Heidelberg, Germany); rabbit monoclonal anti-Ubiquitin (10H4L21) from Life Technologies (Monza, Italy); rabbit polyclonal anti-Caspase3 (#9662) from Cell Signaling (Danvers, MA, USA). The secondary antibodies were: mouse monoclonal anti-mouse IgG1 kappa light chain (#MAB10758) from Millipore (USA) or anti-mouse IgG peroxidase conjugate (A4416) from Sigma-Aldrich (Milan, Italy) or goat anti-rabbit IgG (H + L)-HRP Conjugate (#1706515) from Bio-Rad.

### Generation of a rabbit polyclonal anti-human PON2 antibody

To analyze by mass spectrometry the PON2 PTMs we firstly attempted, without success, quantitative IP of PON2 from HeLa crude extracts with the monoclonal antibody (C-5, sc-374158 from Santa Cruz Biotechnology, Heidelberg, Germany) under native or denaturing conditions. The anti-PON2 raised against amino acids 61–113 mapping within an internal region of human PON2 is unable to efficiently immunoprecipitate PON2 under our conditions. A rabbit polyclonal anti-PON2 antibody was generated by Covalab by using the recombinant PON2 expressed and purified from *E. coli* by us, as described^18^. Four pre-immune bleeds from four different rabbits were tested in our lab to select the most suitable hosts. After a 67-days protocol, the final serum was purified by Covalab on Protein A Sepharose column.

### PON2 IP

HeLa cells (2 × 10^7^) were solubilized in lysis buffer. A total amount of 500 µg of proteins from the soluble fraction was diluted in RIPA buffer (25 mM Tris–HCl pH 7.4, 150 mM NaCl, 1% Nonidet P-40, 0.5% sodium deoxycholate, 0.1% SDS) and then incubated overnight with 2 µg of anti-Ubiquitin or anti-PON2 at 4 °C under rotary agitation. To reduce non-specific binding and background, 40 µl of protein A-coupled Sepharose beads (Sigma-Aldrich) were incubated for 1 h with 0.1% of BSA, washed with PBS, and equilibrated in RIPA buffer. The pre-blocked protein A beads were added to the mixture (total proteins + antibody) at 4 °C for 4 h under rotary agitation. After incubation, the beads were washed with lysis buffer and the protein eluted with Laemmli buffer. In parallel, as a negative control, the soluble fraction was directly incubated with the Protein-A-to exclude any specific binding of the protein to the Protein A-beads. The eluted proteins were analyzed by western blot analyses with the anti-PON2 antibody.

### RNA–protein pull-down

Streptavidin-coated beads were used to purify proteins interacting with the conserved UUUCCUUAAAAU sequence. Briefly, a 30 ribo nt RNA oligo (100 pmol) corresponding to the WTAP 3′ UTR region, with phosphorothioate bonds and biotinylated at 5′ (Eurofins Genomics, Italy), was bound to beads (50 μl) in 500 μl binding buffer (10 mM Tris–HCl, pH 8.0, 1 mM EDTA, 0.25 M NaCl, 0.5% (v/v) TritonX-100), for 30 min at room temperature in a rotating wheel. The supernatant was removed and beads were washed three times with the binding buffer to eliminate unbound oligos. Then the bead–RNA complexes were collected to the bottom of a tube by centrifugation at 1500 × *g* in a microcentrifuge. The pellets were resuspended in HeLa cells protein extracts (500 μg), 1x protease inhibitor (Roche, Monze, Italy) in binding buffer plus 50 mM KCl, 2.5 mM MgCl_2_ and 0.25% Nonidet P-40 (final volume 500 μl). Incubation for 2 h at room temperature in a rotating wheel followed. A control was prepared by adding 1 nmol free oligo as a displacer. Beads were washed three times with five volumes of binding buffer and left for 5 min at room temperature. The proteins were eluted in SDS buffer and resolved by SDS–PAGE.

**Fig. 1:**
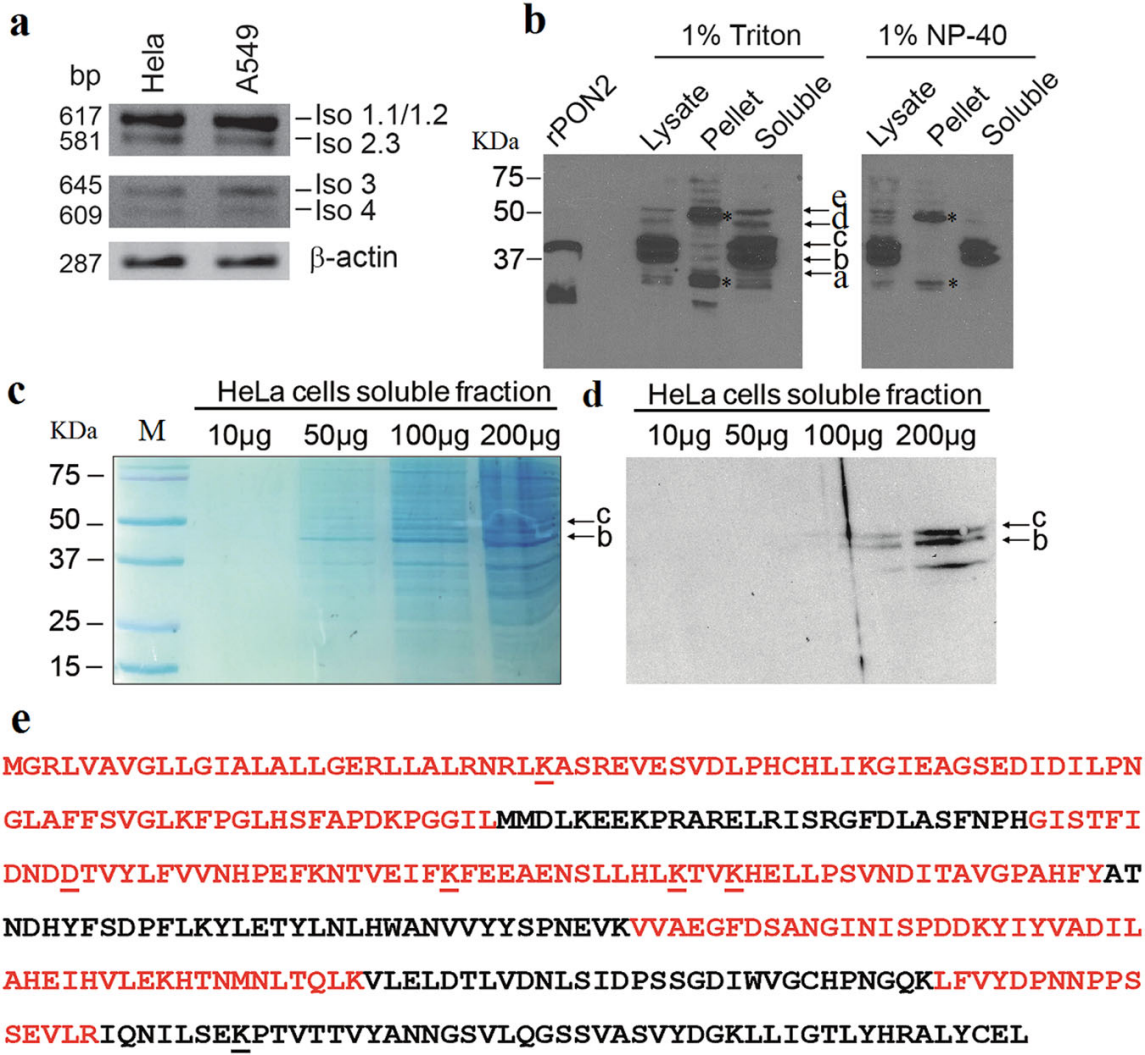
PON2 presents different isoforms (RNA and proteins) in HeLa cells. **a** Reverse trascription-PCR analysis performed with properly designed primers (Supplementary Table 1) in HeLa and A549 cells showing the RNA isoforms of PON2. **b** Western Blot analysis with anti-PON2 antibody of HeLa cells detergents-treated soluble extracts. rPON2 is a purified recombinant PON2 from insect cells (44 kDa) used as control. Lanes to the right of the control are equal amount of lysate, pellet and soluble fraction obtained from treatment with 1% Triton or Nonidet P-40 as indicated. Major bands are indicated as a–e. Asterisks indicate bands enriched in pellets. **c** Coomassie-stained gel of indicated increasing amount of HeLa cells soluble fraction showing the two isoforms of PON2 used in-gel trypsin digestion for MS analyses. **d** Western Blot analysis with anti-PON2 antibody of increasing amount of HeLa cells soluble fraction as in panel **c**. **e** Sequence coverage by MS (in red) of the naive PON2 Isoform 1 from HeLa cells.

### Trypsin digestion and Liquid chromatography–mass spectrometry (LC–MS/MS) analysis

#### Tryptic in-gel digestion

Upon separation by SDS–PAGE and staining with Coomassie Brilliant Blue, protein bands were excised from gels and subjected directly to tryptic digestion. The gel slices were de-stained by 1-h incubation in 50 mM NH_4_CO_3_ containing first 10% and then 50% acetonitrile followed by final incubation in 100% acetonitrile, until band dehydration. The bands were dried under vacuum. The de-stained slices were incubated at room temperature for 1 h in 20 µl of 0.025 µg/ml of trypsin (Trypsin Gold, Mass Spectrometry Grade, Promega, Milan, Italy) in 40 mM NH_4_CO_3_/10% acetonitrile. An additional 50 µl of 40 mM NH_4_CO_3_/10% acetonitrile was added and the slices were digested overnight at 37 °C. The supernatant containing peptides mixtures were collected and dried under vacuum. The dried material was rehydrated in 0.1% formic acid (FA)/2% acetonitrile/water and opportunely diluted before LC–MS/MS analysis.

### LC–MS/MS analysis

Mass spectrometry was performed on a Triple Quadrupole TOF mass spectrometer (5600^+^, AB Sciex, Framingham, MA, USA) equipped with an Eksigent NanoHPLC, a C18 capillary column (AB Sciex) and a nano source (AB Sciex nanospray). The HPLC system was comprised of a solvent degasser, a binary pump and 0.1% FA in 2% acetonitrile and Phase B consisted of 0.1% FA, 2% water in acetonitrile. From the temperature-controlled micro-autosampler, 1–3 µl of the tryptic peptide mixture was automatically loaded onto the column (C18, 3 μm diameter 120 Å) with the binary pump at a flow rate of 250 nL/min (100% Phase A). The peptides were eluted from the column with a 120 min gradient as follows: from 10% to 25% Phase B in 25 min; from 25% to 50% in 25 min; from 50% to 90% in 15 min; isocratic 90% Phase B for 10 min and back to 10% in 15 min at a constant flow rate of 250 nl/min. MS/MS spectra were analyzed by Mascot server Release 2.5^33^ (available at http://www.matrixscience.com/search) or by Paragon Plus algorithm Release 5.0 (AB Sciex)^34^.

### Mutagenesis of 123–134del rPON2, S311C rPON2, and A148G rPON2

Starting from the pT7-7-*hPON2* L52F/K53N/A54V/S55H/E59T/S60P construct^18^, we used the QuikChange Lightning Site-Directed Mutagenesis Kit (Agilent Technologies, Santa Clara, CA, USA), following the manufacturer’s instructions. To obtain 123-134del rPON2, by removing 12 amino acids from the residue 123 to 134 we used as mutagenic primers the following complementary pairs of oligonucleotides:

*PON2-123-134del* forward 5′-actttcatagacaac-gaattcaagaatacagtgg-3′;

*PON2-123-134del* reverse 5′-tattcttgaattc-gttgtctatgaaagtgct-3′.

For the mutant rPON2 S311C we have mutagenized the residue 311 into C and used as mutagenic primers the following complementary pairs of oligonucleotides:

*PON2-S311C* forward 5′-gcatccagaacattctatgtgagaagcctacagtga-3′;

*PON2-S311C* reverse 5′-tcactgtaggcttctcacatagaatgttctggatgc-3′.

For the mutant A148G rPON2 by mutagenizing the residue 148 into G, were used the following complementary pairs of oligonucleotides:

*PON2 A148G* forward 5′ -atttttaaatttgaagaaggagaaaattctctgttgcat-3′

*PON2 A148G* reverse 5′ –atgcaacagagaattttctccttcttcaaatttaaaaat-3′.

### Expression, refolding, and purification of enzymes

Protein expression, in vitro refolding, and purification of rPON2, 123–134del rPON2, S311C rPON2, and A148G rPON2 were performed as previously published in Mandrich et al.^18^ with minor modifications. For the purification of 123-134del rPON2, Sr311C rPON2, and A148G rPON2 the protocol has been slightly modified, using only 2.5 ml of Ni-NTA resin from Qiagen (Hilden, Germany) and by adding 10 mM imidazole in the washing steps before elution of the fractions with buffer B (20 ml) containing 150 mM imidazole. These modifications were introduced because during the other purification steps mutated rPON2 have shown the presence of more contaminants.

### Enzyme assays

The time course of the catalyzed hydrolysis of paraoxon, methyl-paraoxon, parathion, methyl-parathion, coumaphos, malathion, dursban, diazinon, *p*NP-esters, and bis-*p*NP-phosphate (B*p*NP-P), was monitored as described^35,36^. The catalytic activity on different substrates was analyzed by spectrophotometer assay, in which the *p*-nitrophenoxide production after the hydrolysis of the substrates was recorded. The rate of hydrolysis was followed by monitoring the change in absorption at 405 nm (the wavelength at which there is the absorption maximum of the *p*-nitrophenol), using a Cary 100 double-beam spectrophotometer (Varian, Palo Alto, CA, USA) that automatically subtracted blanks.

Standard assays were performed at 40 °C, in a mixture of 20 mM Hepes buffer pH 8.5/0.5 mM CaCl_2_/4% acetonitrile, containing *p*NP-propionate (100 μM). The absorption coefficients at 405 nm used for *p*-nitrophenoxide were 20,000 M^−1^ cm^−1^ at 40 °C (pH 8.5). Initial velocities versus substrate concentration (0.025–2.0 mM) data were analyzed with the GRAFIT program (Grafit Version 3.0, Erithacus Software Ltd., UK). Kinetic parameters on TBBL (5-thiobutyl-γ-butyrolactone) were measured as reported^37^. Assays were carried out in duplicate or triplicate, and the results for the kinetic data were the average of two independent experiments.

Enzyme activity toward homoserine lactones was measured by pH-titration, using a pH-stat apparatus (T50 titrator model, Mettler Toledo, USA). The assays have been performed at 25 °C, in 5 mL water adjusted to pH 8.5 with NaOH 0.1 M, containing 2% acetonitrile and 0.5 mM CaCl_2_. Stock solutions of 3OC12-HSL were prepared by dissolving pure lactones in acetonitrile. Kinetic parameters were measured with substrate concentrations ranging from 0.1 to 1.2 mM; for each point, the blank was measured and subtracted. Assays were performed in technical duplicate or triplicate and results are the averages of at least two independent experiments. Data of initial velocity as a function of substrate concentration were analyzed by the linearization of the Lineweaver–Burk equation for Michaelis–Menten equation, using the GRAFIT program.

### SAXS data collection and processing

Samples were dialyzed against a buffer containing 10 mM HEPES and 0.5 mM CaCl_2_ at pH 8.5 immediately before use. After dialysis, each sample was extensively centrifuged to remove most of the possible aggregated proteins, and the concentration of the protein in the sample was determined by measuring the absorbance at 280 nm. Glycerol (2%) was added immediately before data collection as a free radical scavenger to minimize the radiation damage of the sample.

Small-angle X-ray scattering spectra of 0.75 mg/ml of wtrPON2 and 1.23 mg/ml of deletion mutant 123–134delrPON2 were collected at the BL11 NCD beamline at the ALBA synchrotron (Barcelona, Spain). The sample-detector distance of 2.963 m covered the range of momentum transfer 0.01 < *s* < 3.5 nm^−1^ (*s* = 4*π* sin (*θ*)/*λ*, where 2*θ* is the scattering angle and *λ* = 0.0999 nm is the X-ray wavelength; the optical path of the X-ray through the sample is about 1.2 mm). Samples were kept at 20 °C, and data were collected using a CCD detector. For each sample, we recorded 20 spectra of 1 s each, for a total of 20 s of acquisition. The comparison of the 20 next exposures of an acquisition experiment indicated no changes in the scattering patterns, i.e., no measurable radiation damage to the protein samples. Acquired scattering images were radially integrated with a python script using the pyFAI library^38^. Data were normalized to the intensity of the transmitted beam, and the scattering data from the dialysis buffer (identical to that of the sample) without protein, recorded before and after each corresponding sample measure, were averaged and used to subtract the background. Subtracted spectra were normalized by the protein concentration.

We processed the data using standard procedures for ATSAS programs^39–41^. Data fitted with the Guinier approximation were used to evaluate the gyration radius, *Rg*, and the forward scattering *I*(0) (for *q* < 1.3*Rg*)^42,43^. Sample molecular mass, MM, was estimated by comparing the extrapolated forward scattering *I*(0) to reference solutions of β-amylase, alcohol dehydrogenase, bovine serum albumin, and carbonic anhydrase^44^. The pair distance distribution function *P*(*r*) was calculated using the indirect Fourier transform method implemented in the program GNOM^45^. It was limited to *q* ≤ 2.0 nm^−1^, to avoid the high noise found for larger *q*. The pair distance distribution function also allows determining the maximum size of the scattering particle, Dmax. A Kratky plot was used to qualitatively assess the overall conformational state of the protein^46^.

### Ab initio structural modeling of the SAXS data

Low-resolution models of the PON2 constructs were reconstructed ab-initio using the program DAMMIF^47^ and DAMMIN^48^. These programs represent the protein as an assembly of beads inside a defined search volume of diameter *Dmax*. Starting from a random assembly, DAMMIF employs simulated annealing to build scattering equivalent models fitting the experimental data *I* exp(*q*) to minimize discrepancy:

$$\chi ^2 = \frac{1}{{N - 1}}\left[ {\frac{{I_{\exp }(1_j) - \zeta I_{{\mathrm {calc}}}(q_j)}}{{\sigma (q_j)}}} \right]$$

where *N* is the number of experimental points, *ζ* a scaling factor, and *I*_calc_(*q*_*j*_) and *σ*(*q*_*j*_) are the calculated intensity from the model and the experimental error at the momentum transfer *q*_*j*_, respectively. Ab-initio reconstruction with DAMMIF was repeated 20 times, and resulting models were aligned and averaged using the package DAMAVER^47^. These average models were used for a further ab-initio reconstruction using the more accurate algorithm implemented in DAMMIN to obtain a final plausible molecular model for each PON2 construct. To obtain quantitative estimates of the degree of the conformational heterogeneity of the 123-134del rPON2 we analyzed the SAXS data using an ensemble optimization method (EOM)^49,50^.

### Statistic analysis

For technical replicates (e.g. enzymatic assays) two or three measurements were used. Regarding the biological replicates because in many cases the number of tests was limited to <4 in graphs we generally reported the average value along with the single values (usually three); otherwise, it has been properly specified in the figures’ legends.

## Results and discussion

### PON2 expression in HeLa and A549 cell lines

Aiming at studying PTMs of PON2 we first analyzed the PON2 expression under steady-state conditions by RNA and protein measurements in Hela and A549 cell lines.

By reverse transcription-PCR analyses (Fig. 1a; Supplementary Table 1) we detected four out of the seven mRNA isoforms that have been previously described^51,52^ and schematically reported in Supplementary Fig. 1. On the Nu-SIEVE agarose gel (Fig. 1a) we observed the full-length mRNA Isoform 1 (comprising nine exons) and the Isoform 2 missing, in exon V, of the region corresponding to residues 123–134.

Isoforms 3 and 4, missing the exons I–III^50^, have also been detected (Fig. 1a). Therefore, in both HeLa and A549 cell types, Isoforms 1–4 were present, at least as mRNA. The densitometric evaluation and the data from sequence analysis and details on the nomenclature adopted for these isoforms are given in Supplementary results, Supplementary Fig. 1 and Supplementary Table 2.

To analyze by immunodetection and mass spectrometry the endogenous PON2 protein and its isoforms, proteins were extracted with two detergents: Triton X-100 or Nonidet P-40 (1%, v/v). The anti-PON2 monoclonal antibody revealed at least five bands: a faint band at 37 kDa (a) not always present; a very strong doublet immediately above (b and c); two bands one below (d) and the other (e) above the 50 kDa marker (Fig. 1b). These signals were detected also in the pellets (SDS extracted), but Nonidet P-40 was less efficient. Besides, some additional and slightly different bands were enriched in the pellets up to around 75 kDa (indicated with asterisks). In this work, we focused on the major PON2 bands (b–e) that could correspond to the two PON2 mRNA splicing variants Iso 1/PP and Iso 2 (Supplementary Fig. 1a, b) and their modifications. Both PON2 versions could be glycosylated on N254 and N323 as previously suggested^53–55^. Although anti-PON2 was quite efficient in detecting PON2 by western blot, unfortunately only a few peptides were detected from cutting out immunoprecipitated proteins from SDS–PAGE bands corresponding to PON2 signals. An explanation could be the high number of interactors under native conditions and/or the interaction with the cell membrane, hampering the access of the antibody to the antigen. Consequently, we raised a polyclonal antiserum against the rPON2^18^. Because no significant improvement of IP was observed, we tried the detergents enrichment of PON2 from whole lysates before SDS–PAGE (Fig. 1c). Bands b and c, identified as PON2 by western blot (Fig. 1d), were confirmed to be PON2 isoforms by mass spectrometry analysis. Band c contained, among other proteins, Iso 1/PP, whereas band b contained Iso 2. A band detected at around 30 kDa that could correspond to Iso 3 and/or 4, was not analyzed by mass spectrometry. The complete list of recovered peptides and features related to PTMs is shown in Supplementary Table 3 (see below for further details). The total coverage of naive PON2 sequence was 70% (Fig. 1e).

### PON2 ubiquitination and ADP-ribosylation in intact cells

Recently, we discovered that enzyme inactivation and ubiquitination of K144 occurred after incubation of the engineered canonical Iso 1 (rPON2) made in *E. coli* with an aliquot of crude extract of HeLa cells previously exposed (20 min) to 100 μM 3OC12HSL^18^. The enzyme inactivation behavior resembled that obtained in HEC293T cells for endogenous PON2^14^.

**Fig. 2:**
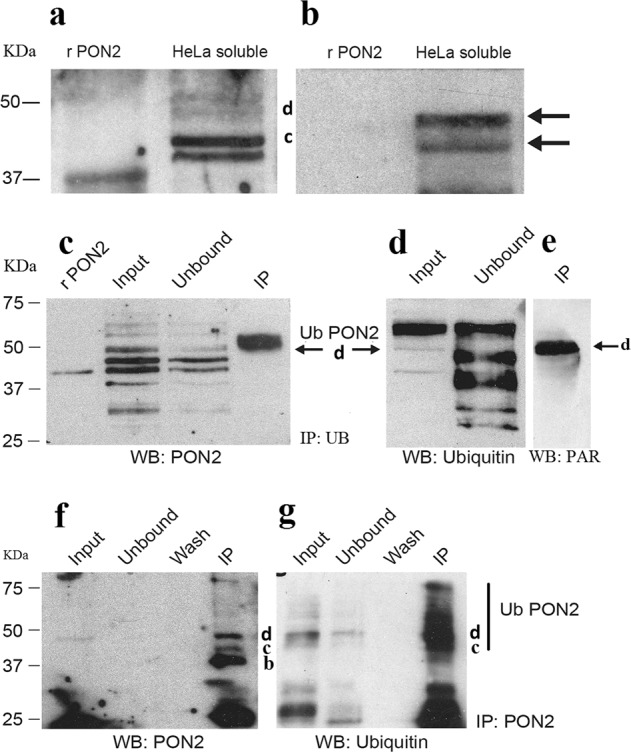
PON2 is ubiquitinated and ADP-ribosylated in intact cells. Western blot analysis with mouse anti-PON2 antibody **a** and with anti-ubiquitin **b** in HeLa cells soluble fractions suggesting PON2 ubiquitination in intact cells. An aliquot (50 μg) of crude extracts was loaded on 12.5% SDS–PAGE and transferred to a NC membrane. In **b** the same filter was used after antibody stripping. rPON2 used as control is a purified recombinant PON2 made in *E. coli* (37 kDa). Western blot analyses aimed to detect ubiquitinated and parylated forms of PON2 in a HeLa soluble fraction used as input after IP using a mouse anti-Ubiquitin antibody. rPON2 used as a control here is a purified recombinant PON2 from insect cells (44 kDa). An equal volume of input, unbound, and immunoprecipitated proteins were loaded and detected with anti-PON2 **c** anti-Ubiquitin **d** and anti-PAR antibodies **e**. A control in which ubiquitin antibodies were omitted was also performed (Supplementary Fig. 2a, b). Western blot analysis of PON2 immunoprecipitated with rabbit polyclonal anti-PON2 antibody (see “Materials and methods” section). An equal volume of input, unbound, wash and immunoprecipitated proteins was loaded and detected with anti-PON2 antibody **f** or with anti-Ubiquitin antibody **g**. Here, in order to avoid the potentially confusing effect of IgG heavy chain (50 kDa), the presence of PON2 was revealed by using an anti-kappa light chain secondary antibody.

To examine whether the same PTM occurs also in living cells, we firstly analyzed by western blot the ubiquitination of endogenous PON2. The NC membrane incubated with rabbit anti-PON2 or anti-Ubiquitin revealed coincident bands (Fig. 2a, b). Then proteins from an IP experiment with a mouse anti-Ubiquitin were analyzed by western blot with anti-PON2 or anti-Ubiquitin antibodies (Fig. 2c, d). A PON2 band below 50 kDa (band d) was enriched (Fig. 2c). This band depleted in the unbound fraction had a relative molecular mass (*M*_r_) around 46–48 kDa and it could be derived from the addition of an Ubiquitin moiety (8.5 kDa) to the 38–40 kDa band. The western blot with anti-ubiquitin revealed, as expected, a band in the input fraction at around 46 kDa (Fig. 2d). The material immunoprecipitated with anti-Ubiquitin was also revealed by western blot with anti-PAR antibodies (Fig. 2e). A coincident band was detected in both western blots. Therefore, data strongly suggest that a PON2 form is ubiquitinated and ADP-ribosylated (PARylated) at the same time. In addition, the HeLa extract was immunoprecipitated with monoclonal (mouse) anti-PON2 and again detected by western blot with polyclonal anti-PON2 (Fig. 2f) and anti-ubiquitin (Fig. 2g) antibodies.

Anti-PON2 immunoprecipitated bands b–d of PON2 (Fig. 2f). A strong signal below band b coincides with the band with the asterisk shown in Fig. 1b. The western blot with anti-Ubiquitin (Fig. 2g) allowed to spot a strong signal corresponding to band d and higher ones, particularly one at around 75 kDa (similar to a band enriched in pellets of Fig. 1b). The experiment was different from the previous one since the anti-Ubiquitin antibody immunoprecipitated only the d band (Fig. 2c, d). We confirmed the PON2 ubiquitinated bands seen previously and in addition some higher bands, likely due to poly-ubiquitination and/or additional PTMs (see below).

### Mass spectrometry analysis of PON2 PTMs

We asked if the 3OC12HSL-dependent PON2 ubiquitination seen in the cell-free system^18^ was present in living cells. The bands from crude extracts corresponding to PON2 masses (identified by a western blot duplicate as shown in Fig. 1c) were gel extracted, trypsin digested and identified by nano LC-MS/MS. This approach, appeared more efficient than IP.

We analyzed the mass data from the main bands indicated in the representative Fig. 1c corresponding to the strong doublet (b and c) and band d (Fig. 1b) running between 37 and 50 kDa. In both bands b and c, we identified in addition to the N-terminus of the PON2 canonical Isoform 1 (NP_000296.2), also the following peptide: MGAWGCGLAGDRAGFLGERLLALRNRLK(A), and related ones that correspond to the N-terminus of Isoform PP (AAC41995.1) (Supplementary Tables 2 and 3). This result establishes the expression in cells of the non-canonical isoform that, as said before, was first described as RNA^52^. Furthermore, we were able to detect, for the first time, a peptide demonstrating the existence as protein of the Iso 2. Iso 2 is the one with the deletion of 12 amino acids of exon V and the Nter of Iso 1. The identified peptide is: ELRISRGFDLASFNPHGISTFIDN/EFK, which encompasses the deleted region (Supplementary Table 3 and Supplementary Fig. 9). The 12 residues skipped in Iso 2 on the basis of the PON1 homolog structure^56^ and the PON2 model^18^, enclose H134 of the active site that helps to increase H115 basicity^56^. Therefore, it is highly likely that its deletion makes the protein substantially inactive (see below).

In the lower band (b) of the gel shown in Fig. 1c, we detected the peptide (k)FEEAENSLLHLK(T) (Supplementary Table 3). This peptide contains the polymorphism (SNP) A148G. It has been reported that this SNP in PON2 is related to high fasting plasma glucose, low concentration of HDL cholesterol and type 2 diabetes as well as related cardiac failure problems^57,58^. The lysine we have found ubiquitinated in the previous work^18^ is the one just before the phenylalanine (small-cap, underlined). In a different experiment, we also detected the peptide with a missed trypsin cut: (K)NTVEIFKFEEAENSLLHLK(T). So far, we never detected in HeLa extracts the peptide with the ubiquitinated K144. Instead, we detected the peptide containing the second described SNP, namely S311C (C in this case), but not the ubiquitinated site K313^62^. SNP at 311 has been reported as well to be associated with diabetes and coronary artery disease^59–61^.

Importantly, we detected from band d the following peptide: (K)NTVEIF**K**FEE**A**ENSLLHLk(LRGG) (Table 1). This peptide contains the positions A148 and K144 (in bold) we were interested in. Surprisingly, the rare remnant LRGG residues diagnostic of ubiquitination were not on residue K144 but on residue K156 (underlined). Furthermore, again from band d, we detected an ubiquitination at position K159 in the following peptide: (**K**)FEE**A**ENSLLHLKTVk(GG) (Table 1 and Supplementary Fig. 6).

**Table 1:** Peptides containing the identified PTMs^a^ and comparison with literature.

PON2 (human Iso 1)	Akimov et al. (2018)	This work	Bilan et al. (2017)
K29-ub^c^	–	Iso PP MGAWGCGLAGDRAGFLGERLLALRNRL**k**(A)“GG”(K) Iso 1 MGRLVAVGLLGIALALLGERLLALRNRL**k**(A)“GG”(K)	
K81-ub	LHSFAPD**k**PGGILMM		
D124-ADPrib.	–	(D)ND**d**TVYLFVVNHPEFK(N)^b^ (H)GISTFIDND**d**TVYLFVVNHPEFK(N) (F)NPHGISTFIDND**d**TVYLFVVNHPEFK(N)^b^ (band **c**)	(R)ISRGFDLASFNPHGISTFIDND**d**TVYLFVVNHPEFK(N)
K144-ub	KNTVEIF**k**FEEAENS^b^	(K)NTVEIF**k**FEEAENSLLHLK(T)”GG”(K) (Mandrich et al. 2015)	
K156-ub	ENSLLHL**k**TV**k**HELL	(K)FEEAENSLLHL**k**(T)“GG”(K)^b^ (K)NTVEIFKFEEAENSLLH**k**(T)“LRGG”(K)^b^	
K159-ub	LLHL**k**TV**k**HELLPSV	(K)FEEAENSLLHLKTV**k**(H)“GG”(K)^b^	
K232-ub	INISPDD**k**YIYVADI		
N254-glyc.	LEKHTNM**n**LTQLKVL^b^		
K289-ub	GCHPNGQ**k**LFVYDPN		
K313-ub	IQNILCE**k**PTVTTVY^b^	(R)IQNILCEkPTVTTVY^b^ (with or without 3OC12HSL)	
Y346-p	KLLIGTL**y**HRALYCE^b^		

^a^Data were analyzed by Mascot (Version 2.5 free from the web) and Protein PilotTM (Version 5.0; AB SCIEX) software.

^b^Modification confirmed in a previous work.

^c^Modifications are indicated in bold as follows: ub, ubiquitination; ADPrib, ADP ribosylation; p, phosphorylation.

**Table 2:** Kinetic assays have been performed in 20 mM Hepes pH 8.5, 0.5 mM Ca^2+^, 40 °C with *p*NP-propionate, TBBL, or 3OC12HSL as substrates.

	*k*_cat_ (s^−1^)	K_M_ (mM)	*k*_cat_/K_M_ (s^−1^ mM^−1^)
*Wild type*			
*p*NP-propionate	1.27 ± 0.13	0.90 ± 0.20	1.41 ± 0.32
TBBL	1.10 ± 0.10	0.50 ± 0.15	2.20 ± 0.39
3OC12HSL	4.1 ± 0.4^a^	0.50 ± 0.10	8.20 ± 0.30
*123-134del*			
*p*NP-propionate	0.018 ± 0.001	0.15 ± 0.02	0.12 ± 0.19
TBBL	nd^b^	–	–
3OC12HSL	nd^a^		
*Ser311Cys*			
*p*NP-propionate	0.042 ± 0.001	0.49 ± 0.03	0.085 ± 0.085
3OC12HSL	0.1 ± 0.01^c^		
*Ala148Gly*			
*p*NP-propionate	0.45 ± 0.04	1.60 ± 0.26	0.28 ± 0.25
3OC12HSL	0.45 ± 0.08^c^		

Each point is the main of two independent assays using two different enzyme preparations.

^a^Assayed by pH stat.

^b^nd not detectable.

^c^Activity (U/mg).

**Fig. 3:**
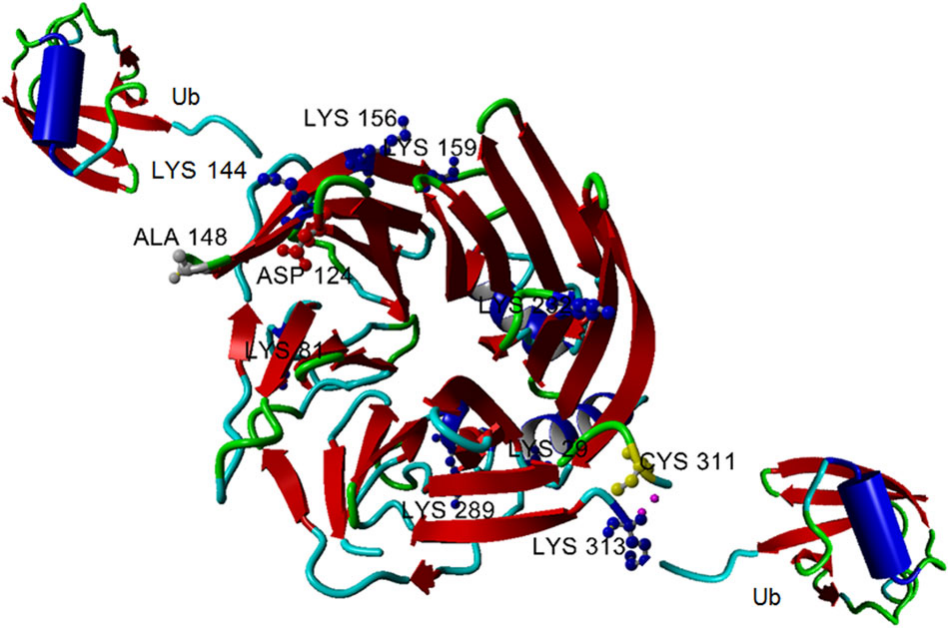
Results of mass spectrometry analyses highlighted on PON2 model. Cartoon of the PON2 model^18^ with highlighted the ubiquitination and the ADP ribosylation sites so far identified (shown in ball and stick representation). The two Ubiquitin moieties (PDB:1UBQ) are attached to the structure only for a demonstrative purpose. The figure was generated with the YASARA program available free of charge at www.YASARA.org. Distances between residues were measured by the same program.

During this work the existence in living cells of the ubiquitination at position K144^18^ was confirmed by Akimov et al. (2018)^63^, which listed seven ubiquitinated peptides of PON2. In addition, the ubiquitination at K156 and K159 that we have found in HeLa cells were confirmed as well. By re-analyzing our old data^18^ the ubiquitination at K156 was observed also in our *cell-free* system. Peptides reported by us in comparison with those listed by others are shown in Table 1. Relevant MS/MS spectra and corresponding table of masses are reported in Supplementary Figs. 4–10. We have also found a new ubiquitination site at position K29 (Table 1 and Supplementary Figs. 4 and 8) in band d (and occasionally in b). The result is interesting for the fact that it belongs with high confidence also to the Iso PP (AAC41995.1). Thus, the coexistence of the PON2 Iso PP and canonical Iso 1 in HeLa cells (see below), is established even with respect to ubiquitination at K29. Akimov et al. (2018)^63^ also detected ubiquitinations at positions K81 and K232 (that we did not detect), but not the one at K29 identified by us (Table 1). In conclusion, we detected three new ubiquitination sites of PON2 in addition to the K144, now confirmed in living cells. In Fig. 3 we highlighted, on the PON2 structural model, the positions of the lysines so far identified as ubiquitinated. As shown, the three lysines K144, K156, and K159 are at 8–12 Å apart. It is worth to recall that the modification at position K144 responds to 3OC12HSL treatment^18^. Furthermore, by highlighting K29 on the PON2 model^18^ it is clear that this position is also nearby to the SNP 311 and the Ub site K313, mirroring in some way the situation at the A148G site. Our working hypothesis is that these lysines are modified depending on physiological or pathological conditions, to modulate the PON2 activity. Support to this idea stems also from the fact that we identified an additional modification in non-treated HeLa cells found also by Bilan et al. (2017)^64^, namely an ADP ribosylation at D124 (see Table 1, Supplementary text and Fig. 10 for additional details). Interestingly, D124 is part of the 12 residues that are not included in the Iso 2 by alternative splicing. By locating this residue on the PON2 model (Fig. 3) the modification seated again nearby the SNP A148G.

### Effect of 3OC12HSL on the ubiquitination of PON2 in HeLa cells

**Fig. 4:**
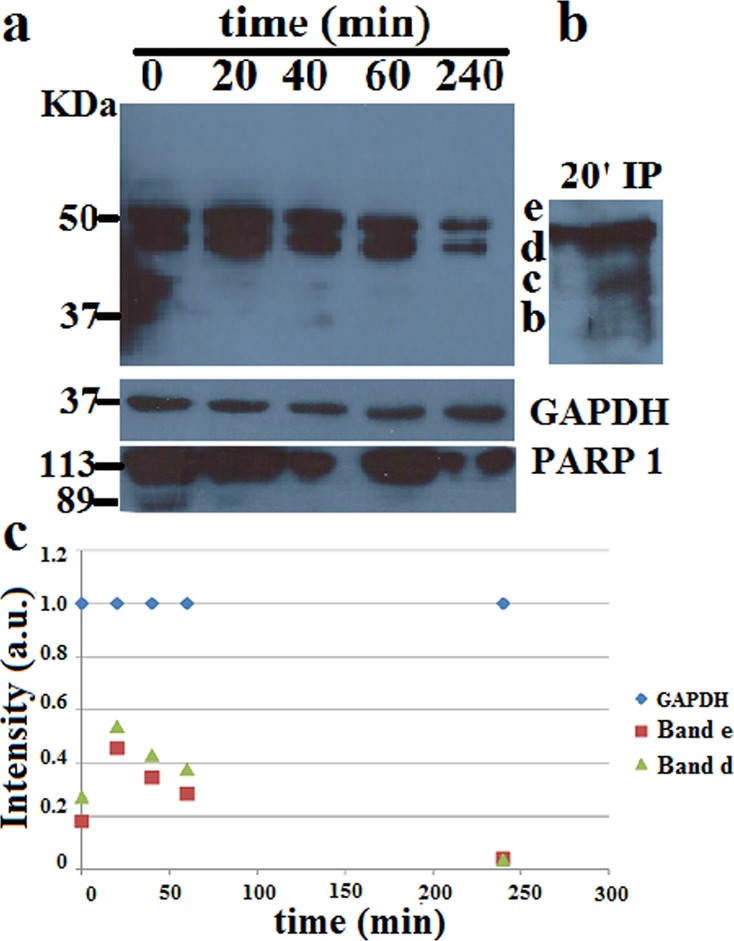
Effect of 3OC12HSL on the ubiquitination of PON2 in HeLa cells. **a** Western blot with anti-PON2 showing the protein behavior in HeLa cells induced with 100 μM 3OC12HSL for the indicated times. GAPDH and PARP-1 fragmentation are also reported **b** IP with anti-Ubiquitin and detection with anti-PON2 of band d after 20 min incubation with 3OC12HSL **c** Densitometric analysis for quantization of band **d**. Data were reported as the average of two or three experiments or single data points as shown.

To assess whether a relation exists between 3OC12HSL treatment and PON2 ubiquitination in living cells we performed a time-course of 3OC12HSL treatment on HeLa. A western blot with anti-PON2 antibody allowed to detect over the time 0–240 min a clear-cut increase of the band d (Fig. 4a, c), already demonstrated to be an ubiquitinated PON2 version (Fig. 2c, d, f, g), with a transient decrease at 10 min (Supplementary Fig. 3a, b). A decrease over longer time incubation (Fig. 4a, c) was observed. The enrichment of band d (with respect to **c** band for example) was confirmed by IP of PON2 with mouse anti-Ubiquitin antibody in cells treated 20 min with 3OC12HSL (Fig. 4b). This experiment confirms that at least one ubiquitination modification is related to bacterial infection.

### Role of PON2 polymorphisms A148G and S311C

**Fig. 5:**
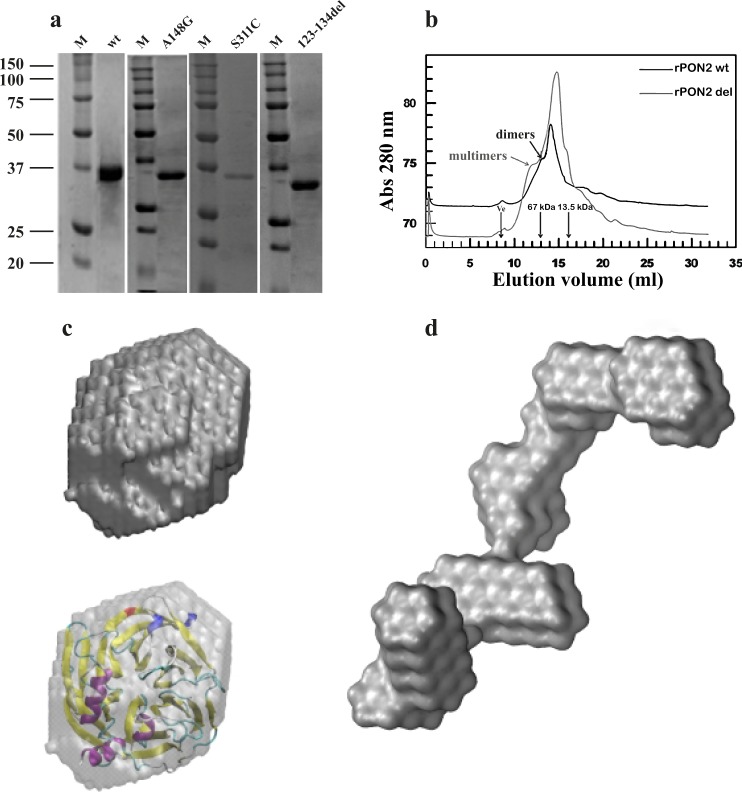
Role of PON2 polymorphisms A148G and S311C. **a** Coomassie-stained gel of recombinant purified PON2 (wild-type), its polymorphic mutants A148G and S311C, and the deletion mutant 123-134del rPON2 produced in *E. coli*; M: molecular markers. **b** Representative gel filtration analysis of rPON2 and 123-134del rPON2 mutants. **c**, **d** SAXS-based reconstruction of the PON2 wt and 123-134del rPON2 structures. The ab initio rigid models for PON2 **c** and 123-134del rPON2 **d**. The structures shown in the figure were drawn at the same spatial scale to compare them. The semi-transparent envelopes in **c** represent the low resolution models of PON2, where the homology atomic structure model has been docked.

Above data on the identification of PON2 PTMs suggest a direct modulating effect on the two SNPs. No data are available in the literature in this respect. Contrasting evidences are present in literature about the effects of the two SNPs^53,54^. Having demonstrated that the rPON2 made in *E. coli* is a good model of the native PON2^18^ (this paper) we decided to test the role of these SNPs on rPON2 activity. We produced and purified (Fig. 5a) as described in “Materials and methods” section, A148G, S311C, and 123-134del rPON2 mutants, the latter corresponding to the Iso 2. The mutations were confirmed by DNA sequencing and by MS for A148G and S311C proteins. Among others, the following peptides were obtained: K.FEEGENSLLHLK.T and R.IQNILCEKPTVTTVYANNGSVLQGSSVASVYDGK.L, confirming the two single mutations.

We measured the kinetic parameters in the wt and the three mutants with an ester (*p*-NP-propionate) and lactones (TBBL and 3OC12HSL) (Table 2). In the deleted PON2 version the esterase activity decreased 70-fold whereas the lactonase activity was undetectable with TBBL and 3OC12HSL (measured by using pH stat).

Circular dichroism spectra in the far and near UV showed similar structures in wt rPON2 and mutants allowing to rule out that the decrease in activity was due to a fault in refolding of mutants (unpublished).

In the S311C and A148G mutants the esterase catalytic activity decreased 30-fold and 3-fold, respectively. However, the specificity decreased 16-fold and 5-fold. The activity against the lactone 3OC12HSL decreased substantially in the S311C and A148G mutants, as for TBBL (Table 2). In conclusion, data demonstrate that the two SNPs and deletion affect heavily the PON2 activity that therefore could be directly involved in type 2 diabetes and related consequences. How and if the lactonase activity and its modulation affect in some way the anti-redox activity remains to be clarified.

### PON2 SAXS structures

Because the deletion of 12 amino acids in the 123-134del rPON2 mutant was predicted to be deleterious for the protein, we decided to approach the resolution of the PON2 structure by SAXS. The wt and 123-134del rPON2 were purified as described in “Materials and methods” section. The last purification steps on gel filtration are shown in Fig. 5b. The deleted mutant showed an elution profile more complex than wt suggesting conformational heterogeneity.

Small-angle X-ray scattering (SAXS) is a method that provides a medium resolution shale pod of macromolecules in solution^43,65^. The physical invariants obtained from SAXS spectra (Supplementary Fig. 11a–f) from solutions containing rPON2 or 123-134del rPON2 are shown in Supplementary Table 4. More technical details and references are given in “Materials and methods” section and Supplementary material. SAXS based ab initio reconstruction of the structure of the wt PON2 was obtained with a resolution of 31.4 Å. The structure reconstruction has a globular shape consistent with the invariants measured directly from the spectra (Supplementary Fig. 11a). We compared this structure with a homology model of the PON2 based on the crystallographic structure of the PON1 (PDB 4Q1U). In Fig. 5c it is clearly observed that the homology model fits well into the reconstructed shape of PON2. Conversely, and in agreement with the data above, the 123-134del rPON2 presents an elongated and multi-lobular shape (Fig. 5d). This shape does not represent a globular protein, but could correspond to a pre-molten protein, or an ensemble of structures occurring in a intrinsically disordered protein (for further details see Supplementary text and Supplementary Figs. 12a–f and 13a–c). In conclusion, SAXS analysis provides evidence that rPON2 is well folded in agreement with enzymatic analysis (Table 2) and detected PTMs, whereas 123-134del rPON2 is unstructured and mostly inactive (in agreement with gel filtration data Fig. 5b). Therefore, it is reasonable to think that the two SNPs lower the PON2 lactonase activity and that PTMs (ubiquitination and ADP ribosylation) observed nearby the SNPs and the skipped region of 12 amino acids are indeed involved in modulation of PON2 activities.

**Fig. 6:**
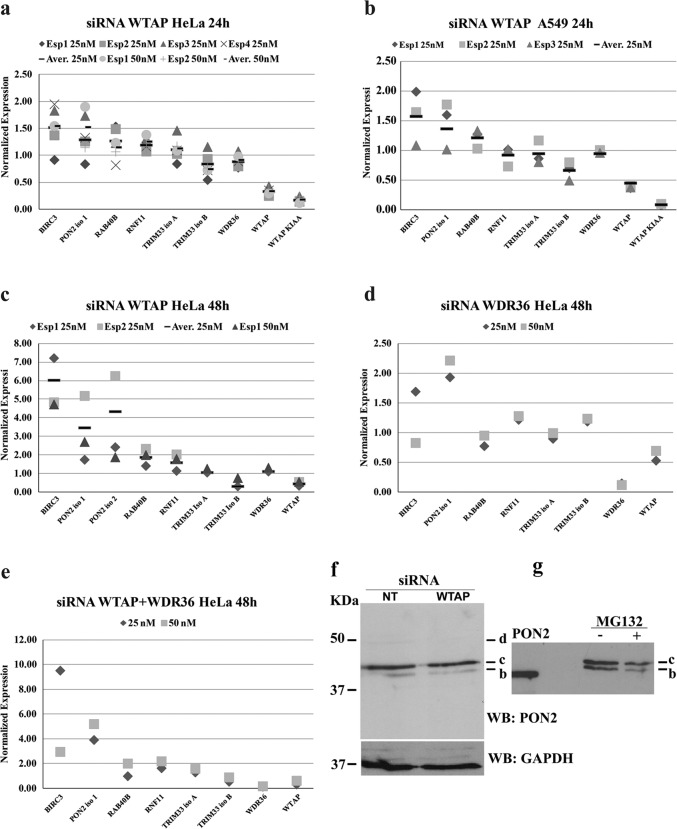
Silencing of the WTAP and WDR36. Expression analysis by quantitative real-time analyses of genes belonging to the “PON2 Cluster” upon the silencing for 24 h of WTAP in the HeLa **a** and A549 **b** cell lines. Expression analysis of the four E3UbLs and the two RBPs belonging to the “PON2 Cluster” upon the silencing for 48 h, respectively, of WTAP **c**, WDR36 **d** and the co-silencing of WTAP and WDR36 **e** in the HeLa cell line. **f** Western blot analysis to determine PON2 expression in WTAP depleted HeLa cells. No-targeting siRNAs (NT) were used as negative control. Total lysate from NT-siRNAs and WTAP siRNAs were electrophoresed and detected with anti-PON2 antibody. **g** Same experiment as in **f** but after treatment of HeLa cells with proteasome inhibitor MG132. Data were reported as the average of three or four independent experiments or single values as shown. Please note that in this experiment the ratio between bands **b** and **c** is slightly different from what observed in Fig. 1b with a predominance of **c** (likely due to extraction or cell culturing conditions).

### The mRNA operon hypothesis

To shed light on the mechanisms of PON2 modifications through functional association by co-expression we tested whether PON2 was co-regulated with the 21 genes of a previously identified cluster^26^ (Supplementary Table 5) sharing a conserved sequence at the 3′ UTRs (Supplementary Table 6). In addition to PON2 (Iso 1 and 2) the more promising genes to analyze were: WTAP, WTAP KIAA (a second isoform of WTAP), and WDR36, because they are both RBPs; TRIM33 Isoforms A and B; BIRC3; RAB40b, and RNF11, all endowed with E3UbL activity. After analyzing the expression level of the selected genes in HeLa and A549 cell lines (Supplementary Fig. 14a) and optimization of silencing conditions (Supplementary Fig. 14b, c), we silenced WTAP, WDR36, or both.

WTAP silencing triggered 1.5-fold up-regulation of BIRC3 and PON2 and 0.75-fold decrease of TRIM33 Iso B in HeLa cells (Fig. 6a). After 48 h silencing, we observed 6–7-fold up-regulation of BIRC3 mRNA (Fig. 6c). In this experiment PON2 mRNA for Iso 1 and 2 increased 3.5 and 4.5-fold, respectively, while TRIM33 Iso B decreased slightly. These data were confirmed in the A549 cell line, in which we observed up-regulation of 1.6-fold and 1.4-fold for BIRC3 and PON2, respectively (at 25 nM for 24 h) and of 0.67-fold for TRIM33 Iso B. RAB40B, RNF11, TRIM33 Iso A, and WDR36 showed minimal variations (Fig. 6b). WDR36 silencing in HeLa cells, produced 1.33-fold up-regulation of PON2 and 0.70 and 0.75-fold down-regulation of RAB40B and WTAP, respectively (Fig. 6d). When cells were knocked down for both WTAP and WDR36 the effect was much more pronounced: PON2 (Iso 1) and BIRC3 increased by 3.9–5.2 fold and 3.0–9.8 fold at 25 and 50 nM, respectively (Fig. 6e).

WTAP silencing was never higher than 65% (Fig. 6b–d), with 85% for WTAP KIAA. This effect could be explained by feedback regulation. The same effect was not observed for WDR36. The fact that WTAP shares the conserved dodecameric sequence at the 3′ UTR could provide a mechanistic interpretation in terms of binding at the 3′ UTR of its own mRNA and modulation of its own splicing, as reported previously^66^.

By analyzing WTAP-silenced cells with monoclonal anti-PON2 the most represented bands b and c were found unchanged with respect to the “no targeting” sample (Fig. 6f). No effects were observed in western blot with anti-PON2 by treating cells with the proteasome inhibitor MG132 (Fig. 6g). Therefore, being the increase of PON2 under WTAP silencing observed at the mRNA level only, there should be something stopping the translation of mRNAs into proteins.

Next we demonstrated that the dodecameric sequence was indeed involved in direct binding of at least WTAP (see “Materials and methods” section, Supplementary results and Supplementary Fig. 15).

From all the above we concluded that at minimum WTAP, PON2, WDR36, and BIRC3 of the cluster form a mRNA operon mediated by the conserved dodecameric sequence of 3’UTR to which at least WTAP binds directly.

### BIRC3 is responsible for PON2 regulation but not ubiquitination

**Fig. 7:**
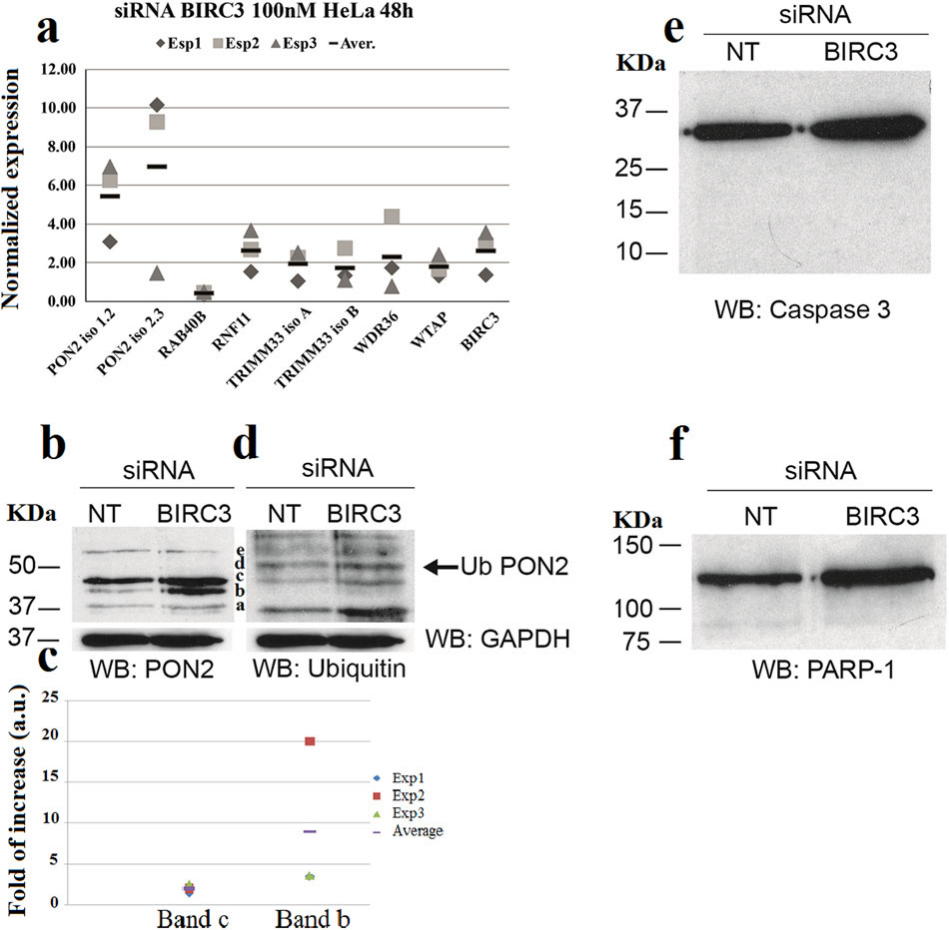
BIRC3 affects PON2 expression level but not its ubiquitination. **a** Expression analysis by quantitative real-time analyses of indicated genes for 48 h in HeLa cell line. Data from single experiments and average are shown as indicated. Western Blot analysis was made to determine PON2 expression and ubiquitination level in BIRC3-depleted HeLa cells. NT-siRNAs were used as negative control. Total lysates from NT-siRNA and BIRC3 siRNA cells were electrophoresed and detected with anti-PON2 antibody **b** and anti-ubiquitin antibody **d**. **c** Densitometric analysis of bands b and c from three different experiments. Data from single experiments and average are shown as indicated. **e** Western blot analysis to determine CASP3 activation in BIRC3-depleted HeLa cells. NT-siRNAs were used as negative control. Total lysates from NT siRNAs and BIRC3 siRNAs-treated cells were electrophoresed and detected with anti-CASP3 antibody. In both lanes is only present the pro-enzyme at 32 kDa in its inactive form. No bands is detected at 17 kDa. **f** Western blot analysis to determine CASP3-mediated PARP-1 cleavage in BIRC3-depleted HeLa cells. NT-siRNAs were used as negative control. Total lysates from NT-siRNAs and BIRC3 siRNAs treated cells were electrophoresed and detected with anti-PARP antibody. Data show only the presence of the enzyme at 113 kDa. No increase is detected for the 89 kDa band.

As already said BIRC3 is an E3UbL involved in apoptosis *via* caspase 3 (CASP3) inhibition^67^. We conjectured that this E3UbL could be involved in PON2 modification. To address this hypothesis we depleted BIRC3 by silencing and analyzed its effect by RT-PCR of the following genes of the cluster: BIRC3, PON2 Iso 1 and Iso 2; RNF11; TRIM33A; TRIM33B; WDR36 and WTAP. By looking at PON2 Iso 1 and Iso 2, we measured 5.5-fold and 7.0-fold increase, respectively (Fig. 7a). Regarding the other genes we observed about two times increase of the others E3UbLs and of the RBPs. The increase of the three E3UbLs could be due to a compensative effect for the BIRC3 silencing. By western blot with anti-PON2 of the “non-targeting” and the BIRC3-depleted cells PON2 Iso 1 increased only two-fold whereas Iso 2 increased more than 10-fold (Fig. 7b, c). By western blot with anti-Ubiquitin there was no apparent change on the ubiquitination level of PON2 (Fig. 7d). In contrast with WTAP silencing, both mRNA and protein increased, but PON2 Iso 2 increased more than Iso 1.

**Fig. 8:**
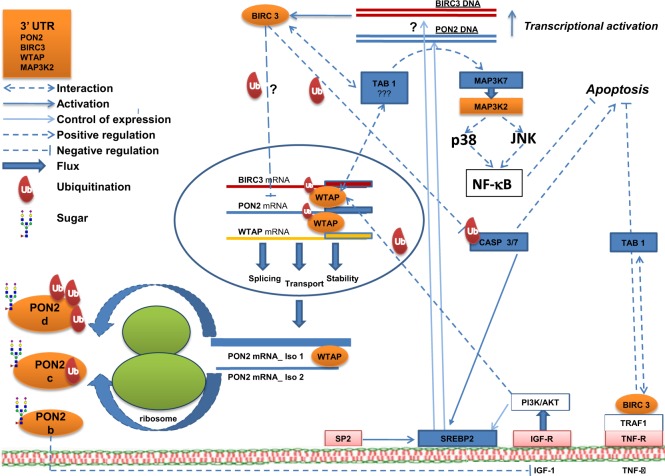
Model of PON2 post-transcriptional regulation. In the model are reported data of protein–protein interactions for WTAP and BIRC3, and their relationship with PON2 as deduced from this paper and literature. Whereas BIRC3 can interact with WTAP *via* TAB1, modulation by ubiquitination it is also possible. WTAP binds a conserved sequence of some mRNAs of the cluster controlling its own splicing (in the nucleus) and stability (in the cytosol)^66^ and those of PON2 and BIRC3 (their mRNA are kept low). BIRC3 in turn controls PON2 splicing (based on our data), likely acting on WTAP, and on translation by acting on SREBP2 (based on chipseq data)^78^. It is worthwhile to notice that BIRC3 inhibits CASP3 activity by mono-Ub^66,76,77^ and that PON2 and BIRC3 both increase following WTAP silencing (this work). CASP3 hyper-activates SREBP2, which in turn activates the expression of PON2 (and maybe of BIRC3 based again on chipseq data)^78^. Therefore, the increase of BIRC3 has a negative feedback effect on itself and on PON2 expression, splicing, and activity. 3OC12HSL stimulates PON2 ubiquitination but BIRC3, however, does not ubiquitinate PON2 in our system.

By continuing exploring the ubiquitination level of PON2, we immunoprecipitated HeLa extracts of silenced or control (non-targeting) cells by anti-Ubiquitin and developed western blots with both anti-PON2 and anti-Ubiquitin antibodies. We confirmed the presence of Ubiquitin signals corresponding mostly to the c and d bands of PON2 (Fig. 7d, e). Thus, whereas it is clear that BIRC3 exerts a negative control on PON2 expression and modulates its alternative splicing (the b band increases 4.8 times as protein), we also excluded that BIRC3 is the E3UbL involved in PON2. It is conceivable that BIRC3 is involved in decreasing PON2 expression via ubiquitination of a yet to be identified factor. About the role of Iso 2, its increase following BIRC3 silencing, should favor a pro-apoptotic role, having BIRC3 an anti-apoptotic function. Since PON2 seems to have a general anti-apoptotic or pro-survival role through its anti-redox activity, we can argue that the Iso 1/PP, endowed with enzyme activities and well-structured, should also have this function more than Iso 2. The corresponding increase of PON2 Iso 2 by RNA and protein also allows to identify with certainty band b as the Iso 2. By looking at the behavior of genes analyzed under BIRC3 silencing (Fig. 7a) we observed a 2.65-fold increase of WTAP. WTAP in addition to being an RBP is also reported to be an adapter protein controlling RNA splicing and RNA methylation^68,69^. Interestingly, WTAP is able to auto regulate its own alternative pre-mRNA splicing and controls also the splicing of baculoviral IAP repeat 5 (BIRC5/survivin)^66^. Therefore, the increase of WTAP following the BIRC3 silencing could be due to autoregulation at the splicing level. It is tempting to speculate that WTAP is the unknown factor modified by BIRC3 and that this is the mechanism by which WTAP (and maybe WDR36) controls the co-expression of PON2 and BIRC3. Accordingly, WTAP and BIRC3 both interact with MAP3K7-binding protein 1 (also known as TAB1)^70,71^, which is a kinase able to bind and activate MAP3K7 and MAP3K2 *via* MAP2K7. MAP3K2 in turn is present in the cluster of proteins sharing the conserved 3′ UTR (Supplementary Table 5) but has not been analyzed in this work. In addition, both WTAP^72^ and BIRC3^73^ have been recently implicated in the severity of glioma suggesting their involvement in the same pathway. PON2 transcriptional expression is under the control of the SREBP2^74^, a transcriptional activator that is released from a membrane precursor upon SP2 hydrolysis^75^. CASP3 does the same job under apoptotic conditions^76^ and because BIRC3 when activated under apoptotic-signaling inactivates CASP3^67,77,78^, we reasoned that the PON2 increase could also be post-transcriptionally modulated by the pathway BIRC3-CASP3-SREBP2. Accordingly, when we analyzed BIRC3-silenced HeLa cells with anti-CASP3 no activation of CASP3 was observed (no band at 17 kDa; Fig. 7e). Anti-PARP1 western blot analysis further confirmed we were not under apoptotic conditions (Fig. 7f). Therefore, our hypothesis is that under normal conditions BIRC3 blocks even small amounts of active CASP3 and this results in reduced SREBP2 activation. If BIRC3 is silenced, even small amounts of CASP3 can hyper-activate SREBP2 that in turn increases the PON2 expression (see Supplementary discussion). We believe that the PON2 increase is regulated under apoptotic conditions by BIRC3 *via* CASP3-SREBP2, whereas its splicing generating Iso 2 is controlled *via* WTAP and interaction of BIRC3 with WTAP *via* TAB1. WTAP, in turn, controls BIRC3 expression (we do not know in which way: splicing as in Birc5?, stability of messenger as for cyclin A2?). Interestingly, SREBP2 could regulate the transcription of BIRC3 as well^79^ (chipseq data at https://cbcl.ics.uci.edu/public_data/SREBP2/). Moreover, in the same data set are present WTAP and MAP3K2. Therefore, the increase of BIRC3 could curb its own expression *via* the same feedback loop CASP3-SREBP2. The model we imagine is shown in Fig. 8 and its legend. For further discussion see Supplementary Information.

## Supplementary information

Supplemental Material

Supplementary Figure 1

Supplementary Figure 2

Supplementary Figure 3

Supplementary Figure 4

Supplementary Figure 5

Supplementary Figure 7

Supplementary Figure 6

Supplementary Figure 8

Supplementary Figure 9

Supplementary Figure 10

Supplementary Figure 11

Supplementary Figure 12

Supplementary Figure 13

Supplementary Figure 14

Supplementary Figure 15

Table S1

Table S2

Table S3

Table S4

Table S5

Table S6

Table S7

Supplementary Figure Legends
