# Supplementary material for: WTAP and BIRC3 are involved in the posttranscriptional mechanisms that impact on the expression and activity of the human lactonase PON2
Source: Cell Death Dis. 2020 May 7;11(5):324. doi: 10.1038/s41419-020-2504-2 (PMC7206036; doi:10.1038/s41419-020-2504-2)
Supplement: Supplementary file 1 — Supplemental Material [file 41419_2020_2504_MOESM1_ESM.docx]

**Supplementary Information for:**

**WTAP and BIRC3 are Involved in the Posttranscriptional Mechanisms that Impact on the Expression and Activity of the Human Lactonase PON2**

**Teresa Maria Carusone^1^, Giovanna Cardiero^2^, Mariangela Cerreta^1^, Luigi Mandrich^1^, Oscar Moran^3^, Elena Porzio^1^, Giuliana Catara^1^, Giuseppina Lacerra^2^*, and Giuseppe Manco^1^***

^1^Institute of Biochemistry and Cellular Biology (IBBC, CNR), National Research Council, Naples, Italy

^2^Institute of Genetics and Biophysics “Adriano Buzzati Traverso”, (IGB-ABT, CNR), National Research Council, Naples, Italy

^3^ Institute of Biophysics (IBF, CNR), National Research Council, Genoa, Italy

***corresponding Authors**

**This ﬁle includes:**

Supplementary text (Introduction, Materials, Results, Discussion, References)

**Supplementary Introduction**

**Role of PON2 in cancer**

Gene expression profiling studies pinpointed the up-regulation of PON2 in some solid tumors, like hepatocellular carcinoma, prostate carcinoma^1,2^ and leukemia^2^. In chronic myeloid leukemia, PON2 was also identified in the gene expression signature of primary imatinib-resistant patients^3^. Referring to the protein, a moderate PON2 over-expression in pancreas, liver, kidney and lung tumors was shown, and an over 10-fold up-regulation in thymus tumors and non-Hodgkins lymphomas^4^.

Therefore, the PON2 anti-apoptotic role may well explain its role in apoptosis and cancer.

However, two recent studies added more complexity to the whole story^5, 6^. Nagarajan *et al*., (2017)^5^ demonstrated the role of PON2 protein/protein interaction in modulating an "old" cancer trait, namely the Warburg effect. In the pancreatic ductal adenocarcinoma cancer (PDAC), the release of p53 translational inhibition of PON2 results in an indirect effect of PON2 on GLUT1, a glucose transporter, mediated by the interaction with the protein stomatin (STOM)^7^. Furthermore, PON2/GLUT1 interaction prevents AMP-activated protein kinase (AMPK)-mediated anoikis *via* ATP inhibition of the pathway AMPK-FOXO3A-PUMA, which in turn favors metastasis^5^. On the other side in the work of Devarajan *et al,* (2018)^6^, the over-expression of PON2 in the early phase of ovarian cancer unexpectedly blocks cancer by reducing IGF-1 production (reduced expression) and its signalling (alteration of IGFR phosphorylation). Intriguingly, IGF-1 expression is due to c-jun under the control of mitochondrial superoxide that is decreased by the anti-redox effect of PON2. Therefore, in this case the PON2 increase, curbs the proliferative effect of IGF-1 and its receptor, becoming anti-tumoral. In line with contrasting effects, in the mitochondria of many cells PON2 is instead forced to fulfil a pro-apoptotic function by releasing lauric acid (C12) after hydrolysis of cell invading bacterial 3OC12HSL. C12 in turn lowers the pH and activates apoptosis^8, 9^.

**Supplementary Materials**

**Table 1**

**Supplementary Results**

The densitometric evaluation and the data from sequence analysis of the four PON2 mRNA isoforms produced similar semi-quantitative results both in HeLa and A549 cell lines. The following relative abundance was determined: Canonical Isoform 1 and/or Primo Parmo version^52^ (Iso1/PP): 60%; Iso1/PP with alternative splicing Exon V (Iso 2): 25%; alternative transcriptional start site (TSS) in the IVSIII (Iso 3): 10%; alternative TSS in the IVSIII plus alternative splicing Exon V (Iso 4): 5% (Fig. 1 a).

**Nomenclature of isoforms**

In the public databases NCBI (https://www.ncbi.nlm.nih.gov/pubmed/), Uniprot (https://www.uniprot.org/) and Ensembl (https://www.ensembl.org/index.html), the analysis of PON2 mRNA and protein isoforms highlighted different nomenclatures and even worse an inconsistency concerning the numbering of isoforms, discordant between mRNA and proteins, as reported in the Supplementary Table 2. To avoid misunderstanding and confusion we decided to use the NCBI nomenclature referred both to mRNA and protein for the isoforms 1 and 2 (Supplementary Fig. 1).

In addition to these two isoforms, noteworthy is the first PON2 isoform characterized by Primo Parmo^10^, classified by NCBI as related sequence with the mRNA code L48513.1 and the protein code AAC41995.1. The sequence of this isoform differs from the canonical sequence in the first 16 amino acids 1-16: MGRLVAVGLLGIALAL → MGAWVGCGLAGDRAGF (Supplementary Table 2).

The L48513.1 mRNA isoform shows conflict: the mRNA sequences of the first exon is not exactly matching with the DNA sequences, showing several differences (Supplementary Fig. 1b). We were not able to understand how this exon could be generated giving rise to the differences in the first 16 amino acids, showed in Supplementary Table 1. We called this isoform as PP (for Primo Parmo; Supplementary Fig 1a). In detail, the Isoform 1 (NM_000305.3, NP_000296.2) corresponds to the “canonical” sequence, namely the full length, 354 aa protein sequence; the Isoform 2 (NM_001018161.1, NP_001018171.1) lacks the peptide ranging from residues 123 to 134 (342 aa; Fig. 1a). The PP Isoform differs from the canonical one for a few different amino acids in the 1-16 aa region. The sequence alignment is shown in Supplementary Fig. 1b. Another anomaly with respect to PON2 mRNAs has been observed for Isoforms 3 and 4; we were not able to identify these isoforms in the public database and this absence is very strange either from the description of Mochizuki H *et al* (1998)^11^, and also because our identifications confirm their presence.

**SAXS analysis**

The molecular mass predicted from the backward scattering extrapolation, *I*(0) (see inserts in Supplementary Fig. 11a and 11b) are consistent with the molecular mass estimated from the amino acid sequence, confirming that the sample was mono-disperse, without aggregates.

The gyration radius, *R_g_* estimated from the Guinier plot for the rPON2 is 20.5 Å, that agrees with the value of 20.5 Å calculated from the inverse Fourier transform of the SAXS spectra. The pair distance distribution function, *P*(*r*) (Supplementary Fig. 11c), is consistent with a quasi-spherical particle with a maximum size, *D_max_,* of 62.4 Å. Conversely, the *R_g_* as calculated from the Guinier plot or the inverse Fourier transform for 123-134delrPON2 are of 42.8 Å and 43.5 Å, respectively. Interestingly, the *P*(*r*) of this isoform presents more than one peak and has a bigger *D_max_* of 141.1 Å, that is consistent with a multi-lobular protein (Supplementary Fig. 11d). The Kratky plot (*q*^2^*I*(*q*) vs *q*)^12, 13^ of the scattering data for the rPON2 is as expected for a globular, compact, protein (Supplementary Fig. 11e), while that of the 123-134delrPON2, corresponds to a multi-lobular, extended, conformation (Supplementary Fig. 11f).

This shape does not represent a globular protein, but could correspond to a pre-molten protein, or an ensemble of structures occurring in a intrinsically disordered protein (Supplementary Fig. 12). In fact, the *R_g_* value is a function of the molecular mass of the molecule, and this function depends on the conformational state of the protein^14^. Thus, while the *R_g_* of the rPON2 corresponds to that expected for a native globular protein, the *R_g_* of the 123-134delrPON2 resembles that expected for a pre-molten molecule, suggesting that this isoform is intrinsically disordered (Supplementary Fig. 12a). Because the Kratky plot, P(r) function, and the dimension of the molecule (*Rg*) indicated that the 123-134delrPON2 is a pre-molten protein, we used an ensemble optimization method (EOM) to quantitatively assess its flexibility. The best EOM solution of ten independent runs yielded very homogeneous χ2 values of ~1.3 (Supplementary Fig. 12b, red line Supplementary Fig. 12c). The best EOM ensemble yielded a set of 6 models for the123-134delrPON2. Single models are presented in the Supplementary Fig. 13a whereas fitting in the DAMMIN reconstruction is shown for comparison (Supplementary Fig. 13b).

**A dodecameric RNA sequence was indeed involved in direct binding of at least WTAP**

A stable (phosphorotioate) and biotinilated RNA oligo was produced and used to affinity precipitate proteins from crude extracts of HeLa cells (see Methods and Supplementary Figure 15*a*). After western blot, only a clear band was detected at around 15KDa (Supplementary Fig. 15b, lane 1). This band disappeared with an excess of competing free oligo (Supplementary Fig. 15b, lane 2). The mass of WTAP is around 50KDa but this protein is heavily ubiquitinated and under certain conditions sent to the proteasome. Therefore we could have detected a more resistant proteolysis fragment.

**Supplementary Discussion**

The emerging picture of the last few years is that PON2 is placed in different districts and exert different functions. Recent studies revealed an emerging link of high PON2 levels with cancer^53^. Therefore, an important issue is how PON2 activities and expression are regulated.  In this study we approached the poorly known regulation of PON2 protein and activity at the post-transcriptional level. Evidence suggests that PON2 is rapidly inactivated by a PTM following the spreading in the cell of 3OC12HSL; this has been observed in vivo^54^ and under cell free conditions^55^. In this work by using Hela cells as a model we have found new PTMs of PON2. We have found some new ubiquitination sites and confirmed older ones. In the band **d** that changed following 3OC12HSL treatment, we found ubiquitinations at positions 29, 156 and 159. In the same band we have also found ADP-ribosylation of D124, under untreated conditions.

Four peptides have been identified in this case among which: (D)N**DdTVYLFVVNHP**EFK, which presented an anomalous cut between D and N and (F)NPHGISTFIDN**DdTVYLFVVNHP**EFK(N) showing as well an anomalous cut between F and N. The shorter peptide was found in the uppermost band (band d) from IP with anti-Ubiquitin antibodies (Fig. 2 c main text) run on a identical gel, whereas the longer peptide in the upper band (band **c**) of the SDS-PAGE not IP (Fig. 1c main text). In the Supplementary Data of Bilan *et al* (2017)^56^ PON2 has been reported as a target of ADP-ribosylation under oxidative stress conditions, namely following the treatment of HeLa cells with 62.5 μM H_2_O_2_. The identified peptide, in that case, was: ISRGFDLASFNPHGISTFID**NdDTVYLFVVNHP**EFK. The long peptide offers an explanation for the fact we detected shorter peptides with non-trypsin cuts, likely due to formic acid cutting head of D^57^ or other nonspecific cuts. The ADP ribosylated residue, also in this case, was D124 (underlined). In the aforementioned work, the peptide was identified after enrichment with the *Archeoglobus fulgidus* macrodomain^56^.

Martello et al, (2016)^58^ by analysing the ADP-ribosylome of intact HeLa cells reported ADP-ribosylation of PON3 but not of PON2. In their Supporting material Bilan et al, (2017) listed an ADP ribosylated peptide modified on D124 under H_2_O_2_ stressing conditions in HeLa cells^56^, but not under normal conditions, as in our case. Therefore, it remains unclear if this ADP-ribosylation may represent or not a response to the oxidative stress. Understanding which enzyme ADP-ribosylates PON2 could help to clarify this point. ADP-ribosyltransferase-5 (ART5), a reported binder of PON2^59^, could be the enzyme catalysing such modification since it is an extracellular enzyme and PON2 is also located on the external side of the plasma membrane^60^. However, a problem is that *in vitro* ART5 displays arginine specificity^61^. Therefore, either *in vivo* ART5 displays a promiscuous aspartate activity or the PON2 ADP-ribosylation reaction is catalysed by another enzyme. The recent comprehensive analysis of the human ADP-ribosylome allowed to point out the extreme infrequency of D specific ADP-ribosylation. In fact, only three peptides were reported to be ADP-ribosylated on D^62^. Due to the D specificity, MARylation of internal PON2 could be catalysed by poly-ADP-ribosyltransferase 10, which actually has substrates localised in the nucleus or mitochondria, where PON2 is localised too^63^. These hypotheses should be tested in the next future.

**PON2 and cancer**

         One of the main characteristics of cancer is resistance to cell death^64^. Knock-down of endogenous PON2 caused spontaneous apoptosis of several human cancer cell lines. Its over-expression lowered susceptibility to different chemotherapeutics in cell culture models. In fact, it has been found that PON2 by diminishing pro-apoptotic mitochondrial O2- formation provides protection against mitochondrial cell death signaling^65^. Accordingly, PON2 deficient HeLa cells exhibit elevated intracellular oxidative level, which can be reversed by over-expression of PON2^66^. PON2 deficiency in mice increases the risk of oxidative stress-related patho-physiological conditions such as the development of atherosclerotic lesions^67,68^. Altenhöfer et al (2010)^69^ demonstrated that PON2 prevents the CoenzymeQ (CoQ)-mediated mitochondrial superoxide generation and apoptosis, independent of its lactonase activity. This points up that the PON2 anti-atherogenic effects are in part mediated by its antioxidative role in mitochondrial function that in turn seems to be based on the binding of CoQ, its subtraction to a ROS-generating reaction^70^, and accordingly high protein expression.

           One mechanistic explanation for high PON2 expression in cancers might be the deregulation of signaling pathways linking reactive oxygen species to cancer^53^. In accordance, earlier studies showed that PON2 expression is enhanced by oxidative stress^71^, and in macrophages, it was induced by cholesterol accumulation and dependent by PPARγ and AP-1 activation^72,73^.

           The urokinase plasminogen activator system may also be significant, as it is increased in a number of cancers, and has been found to up-regulate PON2 again via PI3K/ROS/MEK/ERK/SREBP2 signaling cascade, mediated by the PDGFR-beta and NADPH oxidase activation^70^.

Another important stress and cell death pathway is the unfolded protein response (UPR), which after a recovery attempt, activates apoptosis as a result of insurmountable ER stress. PON2 protects against UPR-mediated apoptosis by negative modulation of JNK signaling, CHOP induction and subsequent CASP3 activation^74^. Therefore, the PON2 anti-redox activity may well explain its role in contrasting apoptosis and facilitating cancer. The increase of PON2 transcriptional activator SREBP2 derives in part from these pathways. However, two recent studies added more complexity to the whole story unrevealing, among others, the role of protein-protein interactions and again of protein expression of PON2 in some cancers^75,76^.

            In this work we hypothesized and confirmed an mRNA operon including at least PON2, BIRC3, WTAP and WDR36. The mRNA operon is a functional unit in which multiple physiologically related transcripts can be co-ordinately regulated during splicing, export, stability, localization, and translation. These subpopulations of mRNAs bind the same RBPs in a dynamic manner because each mRNA can join different RNA operons. Accordingly the operon could work also through the control, mediated by WTAP, of the alternative splicing of its own and controlled genes. BIRC3 inhibits the formation of the PON2 Iso 2 that, according to SAXS structures (that we show here for the first time), seems to encode a partially folded structure, in agreement with the fact that it is 95% inactive in vitro. Although we cannot discharge that the deletion is the cause of a failed renaturation of rPON2, indeed we have also shown that this isoform is expressed in Hela cells as RNA and protein.

           BIRC3 is known to interact with WTAP *via* TAB1^77,78^. Because both BIRC3 and WTAP act on PON2 in a similar way, and BIRC3 does not ubiquitinate the major PON2 isoforms, we hypothesize an indirect effect on PON2 via BIRC3 action on WTAP. WTAP is largely ubiquitinated^79,80^ as also found in our analyses (this work, unpublished). Again this hypothesis requires further work to be confirmed.

**PON2 and diabetes**

Oxidative stress and lipid peroxidation have been indicated as contributors to both the onset and

progression of diabetes and its complications. By-products of the oxidative stress activate several stress pathways such as NF-kB, JNK/SAPK and p38MAP, which fine-tune insulin signaling and contribute to peripheral insulin resistance^81^. As previously mentioned, PON2 exert different roles in order to cope with the oxidative environment. Accordingly, changes in PON2 have been shown to inﬂuence the development of diabetes. Mice lacking the PON2 gene have a greater probability of gaining weight, developing obesity, tolerate glucose in fasting, and show decreased oxygen consumption, energy expenditure and mitochondrial dysfunctions^82^.

The PON2 gene polymorphisms 148 A/G and 311 S/C have been independently associated with

diabetic nephropathy in T2D patients. The susceptibility to diabetic nephropathy was intensiﬁed by the degree of obesity^83^.

In a recent study conducted on 40 Iranian patients with T2D, aged 40–65 years, associated with A allele (SNP PON2-G148A), and C allele (SNP PON2-C311S) it was observed a relationship between PON2 and insulin resistance^84^. In patients with T2D, the daily intake of 2 g of eicosapentaenoic acid (EPA) was shown to increase PON2 expression and decrease glycemic indexes^85^. Pomegranate juice (PGJ) has been shown in some studies to be beneficial for T2D patients^86^. PGJ seed oils are rich in EPA^87^. On the other end PGJ has been shown to increase expression of PON2^72^ .

       In conclusion, we believe to have added more information on the way PON2 is post-transcriptionally regulated, likely under anti-apoptotic conditions and deregulated conditions in cancer, with the involvement of WTAP and BIRC3. It is clear that the full knowledge on how PON2 function is controlled at each level would hopefully open the way in the next future to new therapeutic approaches for diseases such as cancer, diabetes and atherosclerosis where ROS-induced apoptosis is involved and which require control of PON2 expression/activity.

**References**

1. Ribarska T, Ingenwerth, M, Goering W, Engers R. & Schulz, W.A. Epigenetic inactivation of the placentally imprinted tumour suppressor gene TFPI2 in prostate carcinoma. *Can Genom Proteom* **7**, 51-60 (2010).

2. Kang H. et al. Gene expression classifiers for relapse-free survival and minimal residual disease improve risk classification and outcome prediction in pediatric B-precursor acute lymphoblastic leukemia. *Blood* **115**, 1394-1405 (2010).

3. Frank O. et al. Gene expression signature of primary imatinib-resistant chronic myeloid leukemia patients. *Leukemia* **20**, 1400- 1407 (2006).

4. Witte I. et al. Beyond reduction of atherosclerosis: PON2 provides apoptosis resistance and stabilizes tumour cells. *Cell Death Dis* **2**:e112 (2011).

5. Nagarajan A. et al. Paraoxonase 2 Facilitates Pancreatic Cancer Growth and Metastasis by Stimulating GLUT1-Mediated Glucose Transport. *Mol Cell* **67**, 685-701.e6 (2017).

6. Devarajan, A. et al., Paraoxonase 2 deficiency alters mitochondrial function and exacerbates the development of atherosclerosis. *Antioxid Redox Signal* **14,** 341-351 (2011).

7. Havugimana P.C. et al. A census of human soluble protein complexes. *Cell* **150**, 1068-81 (2012).

8. Schwarzer C. et al. Paraoxonase 2 serves a proapopotic function in mouse and human cells in response to the Pseudomonas aeruginosa quorum-sensing molecule N-(3-Oxododecanoyl)-homoserine lactone. *J Biol Chem*. **290**, 7247-58 (2015).

9. Tao S. et al Paraoxonase 2 modulates a proapoptotic function in LS174T cells in response to quorum sensing molecule N-(3-oxododecanoyl)-L-homoserine lactone. *Sci Rep* **6**, 28778 (2016).

10. Primo-Parmo SL, Sorenson RC, Teiber J. & La Du B.N. The human serum paraoxonase/arylesterase gene (PON1) is one member of a multigene family. *Genomics* **33**, 498-507 (1996).

11. Mochizuki H. et al. Human PON2 gene at 7q21.3: cloning, multiple mRNA forms, and missense polymorphisms in the coding sequence. *Gene* **213**, 149-57 (1998).

12. Svergun DI, Koch MHJ, Timmins P.A. & May R. P. Small angle X-ray and neutron scattering from solutions of biological macromolecules. Oxford University Press, 1st edn, vol. **19**, IUCr *Texts on Crystallography*. (2013)

13. Kratky O. & Pilz I. Recent advances and applications of diffuse X-ray small-angle scattering on biopolymers in dilute solutions. *Q Rev Biophys* **5**, 481–537 (1972).

14. Tcherkasskaya O, Davidson E.A. & Uversky V.N. Biophysical constraints for protein structure prediction. *J Proteome Res* **2**, 37–42 (2003).

15. Agricola E, Randall RA, Gaarenstroom T, Dupont S. & Hill C.S. Recruitment of TIF1gamma to chromatin via its PHD finger-bromodomain activates its ubiquitin ligase and transcriptional repressor activities. *Mol Cell* **43**, 85–96 (2011).

16. Nisole S, Stoye J.P. & Saib, A. TRIM family proteins: retroviral restriction and antiviral defence. *Nat Rev Microbiol* **3**, 799–808 (2005).

17. Berndsen C.E. & Wolberger C. New insights into ubiquitin E3 ligase mechanism. *Nat Struct Mol Biol* **21**, 301–307 (2014).

18. Jacob A, Linklater E, Bayless BA, Lyons T. & Prekeris R. The role and regulation of Rab40b-Tks5 complex during invadopodia formation and cancer cell invasion. *J Cell Sci* **129**, 4341-4353 (2016).

19. Piessevaux J, Lavens D, Peelman F. & Tavernier J. The many faces of the SOCS box. *Cytokine Growth Factor Rev*. **19**, 371-81 (2008).

20. Liston P, Roy N, Tamai K, Lefebvre C, Baird S, Cherton-Horvat G, Farahani R, McLean M, Ikeda JE, MacKenzie A. & Korneluk R.G. Suppression of apoptosis in mammalian cells by NAIP and a related family of IAP genes. *Nature* **379**, 349-53 (1996).

21. Gyrd-Hansen M. & Meier P. IAPs: from caspase inhibitors to modulators of NF-kappaB, inflammation and cancer. *Nat Rev Cancer*. **10**, 561-74 (2010).

22. Shembade N, Parvatiyar K, Harhaj N.S. & Harhaj E.W. The ubiquitin-editing enzyme A20 requires RNF11 to downregulate NF-kappaB signalling. *EMBO J.* **28**, 513-22 (2009).

23. Budhidarmo R, Zhu J, Middleton A.J. & Day C.L. The RING domain of RING Finger 11(RNF11) protein binds Ubc13 and inhibits formation of polyubiquitin chains. *FEBS Lett* **592**, 1434-1444 (2018).

24. Cheng J, Yang J, Xia Y, Karin M. & Su B. Synergistic interaction of MEK kinase 2, c-Jun N-terminal kinase (JNK) kinase 2, and JNK1 results in efficient and specific JNK1 activation. *Mol Cell Biol* **20**, 2334-42 (2000).

25. Gallenberger M. et al. Lack of WDR36 leads to preimplantation embryonic lethality in mice and delays the formation of small subunit ribosomal RNA in human cells in vitro. *Hum Mol Genet* **20**, 422-35 (2011).

26. Heo I, Joo C, Cho J, Ha M, Han J. & Kim V.N. Lin28 mediates the terminal uridylation of let-7 precursor MicroRNA. *Mol Cell* **32**, 276-84 (2008).

27. Heo I. et al. TUT4 in concert with Lin28 suppresses microRNA biogenesis through pre-microRNA uridylation. *Cell* **138**, 696-708 (2009).

28. Piskounova E. et al. Lin28A and Lin28B inhibit let-7 microRNA biogenesis by distinct mechanisms. *Cell* **147**,1066-79 (2011).

29. Horiuchi K. et al. Wilms' tumor 1-associating protein regulates G2/M transition through stabilization of cyclin A2 mRNA. *Proc Natl Acad Sci U S A* **103**, 17278-83 (2006).

30. Small T.W. et al. Wilms' tumor 1-associating protein regulates the proliferation of vascular smooth muscle cells. *Circ Res* **99**, 1338-46 (2006).

31. Ortega A. et al. Biochemical function of female-lethal (2)D/Wilms' tumor suppressor-1-associated proteins in alternative pre-mRNA splicing. *J Biol Chem*  **278**, 3040-7 (2003).

32. Jakubauskiene E, Vilys L, Makino Y, Poellinger L. & Kanopka A. Increased Serine-Arginine (SR) Protein Phosphorylation Changes Pre-mRNA Splicing in Hypoxia*. J Biol Chem* **290**, 18079-89 (2015).

33. Wang H.Y. et al. SRPK2: a differentially expressed SR protein-specific kinase involved in mediating the interaction and localization of pre-mRNA splicing factors in mammalian cells. *J Cell Biol* **140**, 737-50 (1998).

34. Tanaka H. et al. A ribonucleotide reductase gene involved in a p53-dependent cell-cycle checkpoint for DNA damage. *Nature* **404**, 42-9 (2000).

35. Tebbi A, Guittet O, Tuphile K, Cabrié A. & Lepoivre M. Caspase-dependent Proteolysis of Human Ribonucleotide Reductase Small Subunits R2 and p53R2 during Apoptosis. *J Biol Chem* **290**, 14077-90 (2015).

36. Chai L, Dai L, Che Y, Xu J, Liu G, Zhang Z. & Yang R. LRRC19, a novel member of the leucine-rich repeat protein family, activates NF-kappaB and induces expression of proinflammatory cytokines. *Biochem Biophys Res Commun* **388**, 543-8 (2009).

37. McGreal EP, Ikewaki N, Akatsu H, Morgan B.P. & Gasque P. Human C1qRp is identical with CD93 and the mNI-11 antigen but does not bind C1q. *J Immunol* **168**, 5222-32 (2002).

38. Wang W. et al NELIN, a new F-actin associated protein, stimulates HeLa cell migration and adhesion. *Biochem Biophys Res Commun* **330**, 1127-31 (2005).

39. Zhu Bet al. Nexilin/NEXN controls actin polymerization in smooth muscle and is regulated by myocardin family coactivators and YAP. *Sci Rep* **8**, 13025 (2018).

40. Tian W. et al. miR663a‑TTC22V1 axis inhibits colon cancer metastasis. *Oncol Rep* **41**, 1718-1728 (2019).

41. Abdel Rahman A.M. et al. Golgi N-glycan branching N- acetylglucosaminyltransferases I, V and VI promote nutrient uptake and metabolism. *Glycobiology* **25**, 225-40 (2015).

42. Leyva-Díaz E. et al. FLRT3 is a Robo1-interacting protein that determines Netrin-1 attraction in developing axons. *Curr Biol* **24**, 494-508 (2014).

43. Liu L. et al. Slit2 and Robo1 expression as biomarkers for assessing prognosis in brain glioma patients. *Surg Oncol* **25**, 405-410 (2016).

44. Manser E. et al. Human carboxypeptidase E. Isolation and characterization of the cDNA, sequence conservation, expression and processing in vitro*. Biochem J.* **267**, 517-25 (2014).

45. Lee T.K. et al. An N-terminal truncated carboxypeptidase E splice isoform induces tumor growth and is a biomarker for predicting future metastasis in human cancers. *J Clin Invest* **121**, 880-92 (2011).

46. Yochum G.S. & Ayer D.E. Pf1, a novel PHD zinc finger protein that links the TLE corepressor to the mSin3A-histone deacetylase complex. *Mol Cell Biol*  **21**, 4110-8 (2001).

47. Bansal N. et al. Targeting the SIN3A-PF1 interaction inhibits epithelial to mesenchymal transition and maintenance of a stem cell phenotype in triple negative breast cancer. *Oncotarget* **6**, 34087-105 (2015).

48. Okumoto K. et al. PEX12, the pathogenic gene of group III Zellweger syndrome: cDNA cloning by functional complementation on a CHO cell mutant, patient analysis, and characterization of PEX12p. *Mol Cell Biol* **18**, 4324-36 (1998).

49. Kao YT, Fleming WA, Ventura M.J. & Bartel B. Genetic Interactions between PEROXIN12 and Other Peroxisome-Associated Ubiquitination Components. Plant Physiol **172**, 1643-1656 (2016).

50. Miyamoto-Sato E. et al. A comprehensive resource of interacting protein regions for refining human transcription factor networks. *PLoS One* **24**;5(2):e 9289 (2010).

51. Letzien U, Oppermann H, Meixensberger J. & Gaunitz F. The antineoplastic effect of carnosine is accompanied by induction of PDK4 and can be mimicked by L-histidine. *Amino Acids* **46**,1009-19 (2014).

52. Li T, Guan J, Li S, Zhang X. & Zheng X. HSCARG downregulates NF-κB signaling by interacting with USP7 and inhibiting NEMO ubiquitination. *Cell Death Dis* **5**, e1229 (2014).

53. Witte I, Foerstermann U, Devarajan A, Reddy S.T. & Horke S. Protectors or Traitors: The Roles of PON2 and PON3 in Atherosclerosis and Cancer. *J Lipids* **2012**, 342806 (2012).

54. Horke S. et al. Paraoxonase 2 is down-regulated by the Pseudomonas aeruginosa quorum sensing signal N-(3-oxododecanoyl)-L-homoserine lactone and attenuates oxidative stress induced by pyocyanin. *Biochem J* **426**, 73-83 (2010).

55. Mandrich L, Cerreta M. & Manco G. An Engineered Version of Human PON2 Opens the Way to Understand the Role of Its Post-Translational Modifications in Modulating Catalytic Activity. *PLoS One* **10**, e0144579 (2015).

56. Bilan V. et al. New Quantitative Mass Spectrometry Approaches Reveal Different ADP-ribosylation Phases Dependent On the Levels of Oxidative Stress. *Mol Cell Proteomics* **16**, 949-958 (2017).

57. Li A. et al. Chemical cleavage at aspartyl residues for protein identification. *Anal Chem* **73**, 5395-402 (2001).

58. Martello R. et al. Proteome-wide identification of the endogenous ADP-ribosylome of mammalian cells and tissue. *Nat Commun* **7**, 12917 (2016).

59. Huttlin E.L. et al. Architecture of the human interactome defines protein communities and disease networks. *Nature* **545**, 505-509 (2017).

60. Hagmann H. et al. Breaking the chain at the membrane: paraoxonase 2 counteracts lipid peroxidation at the plasma membrane. *FASEB J* **28**, 1769-79 (2015).

61. Glowacki G. et al. The family of toxin-related ecto-ADP-ribosyltransferases in humans and the mouse. *Prot Sci* **11**, 1657-70 (2002).

62. Hendriks IA, Larsen S.C. & Nielsen M.L. An Advanced Strategy for Comprehensive Profiling of ADP-ribosylation Sites Using Mass Spectrometry-based Proteomics. *Mol Cell Proteomics* **18**, 1010-1026 (2019).

63. Kaufmann M. & Feijs B. Function and regulation of the mono-ADP-ribosyltransferase ARTD10. *Curr Top Microbiol Immunol*. **384**, 167-88 (2015).

64. Hanahan D, Weinberg RA (2011) Hallmarks of cancer: the next generation. *Cell* 144: 646- 674.

65. Witte I. et al. Beyond reduction of atherosclerosis: PON2 provides apoptosis resistance and stabilizes tumour cells. *Cell Death Dis* **2**, e112 (2011).

66. Ng C.J. et al. Paraoxonase-2 deficiency aggravates atherosclerosis in mice despite lower apolipoprotein-B-containing lipoproteins: anti-atherogenic role for paraoxonase-2. *J Biol Chem* **281**, 29491–29500 (2006).

67. Devarajan A. et al. Paraoxonase 2 deficiency alters mitochondrial function and exacerbates the development of atherosclerosis. *Antioxid Redox Signal* **14**, 341-351 (2011).

68. Ohnishi T. & Trumpower B.L. Differential effects of antimycin on ubisemiquinone bound in different environments in isolated succinate cytochrome c reductase complex. *J Biol Chem* **255**, 3278-84 (1980).

69. Altenhöfer S. et al. One enzyme, two functions: PON2 prevents mitochondrial superoxide formation and apoptosis independent from its lactonase activity. *J Biol Chem* **285**, 24398-24403 (2010).

70.Shiner M, Fuhrman B. & Aviram M. Paraoxonase 2 (PON2) expression is upregulated via a reduced- nicotinamide-adenine-dinucleotide-phosphate (NADPH)-oxidase dependent mechanism during monocytes differentiation into macrophages. *Free Radic Biol Med* **37**, 2052-2063 (2004).

71. Rosenblat M. et al. Mouse macrophage paraoxonase 2 activity is increased whereas cellular paraoxonase 3 activity is decreased under oxidative stress. *Arterioscler Thromb Vasc Biol*  **23,** 468-474 (2003).

72. Shiner M, Fuhrman B. & Aviram M. Macrophage paraoxonase 2 (PON2) expression is up-regulated by pomegranate juice phenolic anti-oxidants *via* PPAR gamma and AP-1 pathway activation. *Atherosclerosis* **195**, 313-21 (2007).

73. Fuhrman B. et al. Urokinase activates macrophage PON2 gene transcription via the PI3K/ROS/MEK/SREBP-2 signalling cascade mediated by the PDGFR-beta. *Cardiovasc Res*  **84**, 145-154 (2009).

74. Horke S. et al. Paraoxonase-2 reduces oxidative stress in vascular cells and decreases endoplasmic reticulum stress-induced caspase activation. *Circulation*  **115**, 2055- 2064 (2007).

75. Nagarajan A. et al. Paraoxonase 2 Facilitates Pancreatic Cancer Growth and Metastasis by Stimulating GLUT1-Mediated Glucose Transport. *Mol Cell* **67**, 685-701.e6 (2017).

76. Devarajan A. et al. Paraoxonase 2 overexpression inhibits tumor development in a mouse model of ovarian cancer. *Cell Death Dis* **9**, 392 (2018).

77. Small T.W. & Pickering J.G. Nuclear degradation of Wilms tumor 1-associating protein and survivin splice variant switching underlie IGF-1-mediated survival. *J Biol Chem* **284**, 24684-95 (2009).

78. Goncharov T. et al. OTUB1 modulates c-IAP1 stability to regulate signalling pathways. **32**, 1103-14 (2013).

79. Beltrao P. et al. Systematic functional prioritization of protein posttranslational modifications. *Cell* **150**, 413-25 (2012).

80. Wagner S.A. et al. C A proteome-wide, quantitative survey of *in vivo* ubiquitylation sites reveals widespread regulatory roles. *Mol Cell Proteomics*. **10**, M111.013284 (2011) .

81. Rains J.L. & Jain S.K. Oxidative stress, insulin signaling, and diabetes. *Free Radic Biol Med* **50**, 567–575 (2011).

82. Shih D.M. et al. PON2 Deﬁciency Leads to Increased Susceptibility to Diet-Induced Obesity. *Antioxidants*, **8**,

19 (2019).

83. Pinizzotto M. et al. Paraoxonase2 polymorphisms are associated with nephropathy in Type II diabetes. *Diabetologia,* **44**, 104–107 (2001).

84. Qujeq D, Mahrooz A, Alizadeh A. & Boorank R. Paraoxonase-2 variants potentially inﬂuence insulin

resistance, beta-cell function, and their interrelationships with alanine aminotransferase in type 2 diabetes.

*J. Res. Med. Sci.,* **23**, 107 (2018).

85. Golzari, M.H. et al. Effect of Eicosapentaenoic Acid Supplementation on Paraoxonase 2 Gene Expression in Patients with Type 2 Diabetes Mellitus: A Randomized Double-blind Clinical *Trial. Clin. Nutr. Res.,* **8**, 17–27 (2019).

86. Banihani S.A. et al. , Fresh pomegranate juice ameliorates insulin resistance, enhances β-cell function, and decreases fasting serum glucose in type 2 diabetic patients, *Nutrition Research*, **34**, 862-867 (2014).

87. Shiner M, Fuhrman B. & Aviram M.. Macrophage paraoxonase 2 (PON2) expression is up-regulated by pomegranate juice phenolic anti-oxidants via PPAR gamma and AP-1 pathway activation. *Atherosclerosis* **195**, 313–321 (2007).
