# Supplementary material for: WTAP and BIRC3 are involved in the posttranscriptional mechanisms that impact on the expression and activity of the human lactonase PON2
Source: Cell Death Dis. 2020 May 7;11(5):324. doi: 10.1038/s41419-020-2504-2 (PMC7206036; doi:10.1038/s41419-020-2504-2)
Supplement: Supplementary file 17 — Table S1 [file 41419_2020_2504_MOESM17_ESM.docx]

| **Table 1. Primers and position of oligos for PON2 mRNA analysis** | | | | | | | | | | |
| --- | --- | --- | --- | --- | --- | --- | --- | --- | --- | --- |
| **Name** | **bp** | **Position** | **Forward primers** | **Name** | **bp** | **Position** | **Reverse**  **primers** | **Assay** | **Ref.** | **Amplicons length (bp)** |
| A | 22 | NM_000305.2  NM_001018161.1  368-389 | GGAGGAATACTAATGATGGATC | B | 22 | NM_000305.2  785-764  NM_001018161.1  749-728 | CTGAATCAAATCCTTCTGCTAC | PCR, sequence | 36 | 418  382 |
| B | 21 | Chr 7  95412639-95412659 | TTTCAGTATTCCACCTCCCCG | B |  |  |  | PCR, sequence | p.a. | 645  609 |
| C | 18 | NM_000305.2  169-186 | CCTGGGCGAGAGGCTTCT | E | 25 | NM_000305.2  525-501 | TCTGGGTGGTTTACAACAAAGAGAT | PCR | p.a. | 356  374  426 |
