# Supplementary material for: WTAP and BIRC3 are involved in the posttranscriptional mechanisms that impact on the expression and activity of the human lactonase PON2
Source: Cell Death Dis. 2020 May 7;11(5):324. doi: 10.1038/s41419-020-2504-2 (PMC7206036; doi:10.1038/s41419-020-2504-2)
Supplement: Supplementary file 18 — Table S2 [file 41419_2020_2504_MOESM18_ESM.docx]

| **Table 2. mRNA and protein RefSeq for the PON2 isoforms from NCBI and Uniprot databases** | | | |
| --- | --- | --- | --- |
| NCBI mRNA | Uniprot | Note from Uniprot | Present code |
| NM_000305.2  Isoform 1  RefSeq  1669 bp | Q15165.4  Isoform 2  Canonical  354aa | This variant represents the longer transcript and encodes the longer isoform | Iso 1.2 |
| L48513.1  Related sequences  1542 bp | Q15165.2  Isoform 1  354aa | *The sequence of this isoform differs from the canonical sequence as follows:* 1-16: MGRLVAVGLLGIALAL → MGAWVGCGLAGDRAGF | Iso 1.1 |
| NM_001018161.1  Isoform 2  RefSeq  1633bp | Q15165.3  Isoform 3  342aa | This variant uses an alternate, in-frame splice site in the coding region, compared to variant 1. (NCBI) *The sequence of this isoform differs from the canonical sequence as follows:* 123-134: Missing. | Iso 2.3 |
