# Supplementary material for: WTAP and BIRC3 are involved in the posttranscriptional mechanisms that impact on the expression and activity of the human lactonase PON2
Source: Cell Death Dis. 2020 May 7;11(5):324. doi: 10.1038/s41419-020-2504-2 (PMC7206036; doi:10.1038/s41419-020-2504-2)
Supplement: Supplementary file 19 — Table S3 [file 41419_2020_2504_MOESM19_ESM.docx]

| **Table 3. List of peptides identified by mass spectrometry analysis in different PON2 bands** | | | |
| --- | --- | --- | --- |
| **Peptides** | **Origin** | **Res.N.** | **Mass** |
| **MGAWVGCGLAGDR** | band(**b**)IP with anti-PON2(polyclonal) and band(**c**)not IP Iso1 PP | 1-13 | 1288.6682 |
| **MGAWVGCGLAGDRM(ox)** | band(**d**)IP with anti-Ubiquitin Iso1 PP | 1-14 | 1306.5463 |
| **MGAWVGCGLAGDRAGFLGERLLAL** | band(**b**)IP with anti-PON2 Iso1 PP | 1-24 | 2429.14 |
| **MGAWVGCGLAGDRAGFLGERLLALRNRLk(GG)** | band(**d**)IP anti-Ubiquitin Iso1 PP | 1-29 | 3212.4231 |
| **WVGCGLAGDRAGFLGERLLALRNRLKASREVE**  **SVDLPHCHLIK** | band(**b**)IP with anti-PON2 Iso1 PP | 1-46 | 4748.3935 |
| **AGFLGERLLALRNRLk(QTGG)** | band(**d**) IP with anti-Ubiquitin (**Ub at 29**) Iso1 PP | 13-29 | 2172.927 |
| **AWVGCGLAGDRAGFLGERLLALRNR** | band(**b**)IP with anti-PON2 Iso1 PP | 3-27 | 2658.6641 |
| **MGRLVAVGLLGIALALLGERLLALRNRLk(GG)** | band(**d**)IP with anti-Ubiquitin (**Ub at 29**)Iso1 canonical | 1-29 | 3212.4231 |
| **MGRLVAVGLLGIALALLGERLLALR** | band(**b**)IP with anti-PON2 Iso1 canonical | 1-25 | 2502.54 |
| **MGRLVAVGLLGIALALLGE** | band(**c**) not IP Iso1 canonical | 1-19 | 1866.3153 |
| **LVAVGLLGIALALLGERLLALRNRLK** | band(**c**) not IP and band(**b**) IP with anti-PON2 Iso1 canonical | 3-29 | 2515.2092 |
| **RLVAVGLLGIALALLGERLLALR** | band(**c**)not IP Iso1 canonical | 3-25 | 2401.2053 |
| **.GRLVAVGLLGIALALLGERLLALR** | band(**c**)non IP Iso1 canonical | 2-25 | 2456.3049 |
| **RLVAVGLLGIALALLGERLLALRNRLk(GG)** | band(**b**)IP with anti-PON2 Iso1 canonical | 3-29 | 3027.3533 |
| **EVESVDLPHCH** | band(**b**)IP with anti-PON2 | 33-43 | 1263.6167 |
| **ASREVESVDLPHCHLIK** | band(**b**)IP with anti-PON2 | 30-46 | 1931.5731 |
| **EVESVDLPHCHLIK** | band(**b**)IP with anti-PON2 | 33-46 | 1618.7261 |
| **GIEAGSEDIDILPNGLAFFSVGLK** | band(**b**)IP with anti-PON2 | 47-70 | 2461.807 |
| **GLAFFSVGLK** | band(**d**)IP with anti-Ubiquitin | 61-70 | 1037.5479 |
| **FPGLHSFAPDKPGGIL** | band(**b**)IP withanti-PON2 K81 not Ub here | 71-86 | 1652.5692 |
| **FPGLHSFAPDKPGGILMMDLK** | band(**d**)IP with anti-Ubiquitin | 71-91 | 2270.5112 |
| **ELRISRGFDLASF**  **NPHGISTFIDN____________EFK** | band(**b**)IP with anti-PON2(polyclonal)(**Iso 2!**) | 111-137 | 3091.6794 |
| **NDdTVYLFVVNHPEFK** | band(**d**)IP with anti-Ubiquitin(**ADPrib in D124)** | 134-149 |  |
| **GISTFIDNDdTVYLFVVNHPEFKNTV** | band(**c**)not IP (**ADP rib in D124**) | 115-140 |  |
| **GISTFIDNDdTVYLFVVNHPEFK** | band(**c**)not IP (**ADP rib in D124**) | 115-137 |  |
| **NPHGISTFIDNDdTVYLFVVNHPEFK** | band(**c**)not IP (**ADP rib in D124**) | 112-140 | 3560.579 |
| **GISTFIDNDDTVYLFVVNHPEFK** | band(**b**)IP with anti-PON2  band(**c**) and (**b**) not IP | 115-137 | 2670.921 |
| **NTVEIFK**  **FEEAENSLLHLk(LRGG)** | band(**d**)IP with anti-Ubiquitin (**Ub at 156**) | 138-159 | 2643.1921 |
| **FVVNHPEFKNTVEIFK**  **FEEAEN** | band(**b**)IP with anti-PON2 | 129-150 | 2669.32 |
| **NTVEIFK** | band(**b**)IP with anti-PON2 | 138-144 | 850.9653 |
| **FEEAENSLLHLKTVk(GG)** | band(**d**)IP with anti-Ub and (**b**) IP with anti-PON2 (**Ub at 159**) | 145-159 | 1872.85 |
| **FEEAENSLLHLK** | band(**b**)IP with anti-PON2 and not IP | 145-156 | 1428.729 |
| **FEEAENSLLH** | band(**b**)IP anti-PON2 | 145-154 | 1187.0231 |
| **HELLPSVNDITAVGPAHFY** | band(**d**)IP anti-Ubiquitin | 160-178 | 2080.1201 |
| **HELLPSVNDITAVGPAHFYATNDHYFSDPFLK** | band(**b**)IP with anti-PON2 and band (**c**) not IP | 160-191 | 3614.6958 |
| **VVAEGFDSANGINISPDDK** | band(**b**) not IP and band (**d**) IP with anti-Ubiquitin | 214-232 | 1946.5746 |
| **YIYVADILAHEIHVLEK** | band(**b**)IP with anti-PON2  band(**b**)notIP and band (**d**) IP with anti-Ubiquitin | 233-249 | 2026.334 |
| **DILAHEIHVLEK** | band(**b**)IP with anti-PON2 | 238-249 | 1414.4841 |
| **LFVYDPNNPPSSEVLR** | band(**c**)not IP and band (**d**)IP with anti-Ubiquitin | 290-305 | 1844.93 |
| **FVYDPNNPPSSEVLR** | band(**d**)IP with anti-Ubiquitin | 291-305 | 1733.2362 |
| **VYDPNNPPSSEVLR** | band(**d**)IP with anti-Ubiquitin and band(**b**)IP with anti-PON2 | 292-305 | 1583.8479 |
