# Supplementary material for: WTAP and BIRC3 are involved in the posttranscriptional mechanisms that impact on the expression and activity of the human lactonase PON2
Source: Cell Death Dis. 2020 May 7;11(5):324. doi: 10.1038/s41419-020-2504-2 (PMC7206036; doi:10.1038/s41419-020-2504-2)
Supplement: Supplementary file 20 — Table S4 [file 41419_2020_2504_MOESM20_ESM.docx]

| **Table 4.** Physical invariants of the rPON2 and 123-134delrPON2 as measured from the SAXS data | | |
| --- | --- | --- |
|  | **rPON2** | **123-134delrPON2** |
| *R_g_* (nm) | 2.07 ± 0.04 | 4.28 ± 0.09 |
| *I*(0) | 4.3 ± 0.88 | 4.05 ± 0.98 |
| *R_g_** (nm) | 2.05 | 4.35 |
| *D_max_* (nm) | 6.24 | 14.11 |
| *MM_calc_* (kDa) | 37.69 | 36.29 |
| *MM_exp_* (kDa) | 35.95 ± 1.97 | 33.86 ± 1.89 |

*R_g_* : gyration radius, Guinier approximation; *I*(0): backward scattering intensity, Guinier approximation; *R_g_**: gyration radius, calculated from the inverse Fourier transform; *D_max_* : maximum size of the particle; *MM_calc_* and *MM_exp_* molecular mass, calculated from the protein sequence and estimated from *I*(0).
