# Supplementary material for: WTAP and BIRC3 are involved in the posttranscriptional mechanisms that impact on the expression and activity of the human lactonase PON2
Source: Cell Death Dis. 2020 May 7;11(5):324. doi: 10.1038/s41419-020-2504-2 (PMC7206036; doi:10.1038/s41419-020-2504-2)
Supplement: Supplementary file 21 — Table S5 [file 41419_2020_2504_MOESM21_ESM.docx]

| **Table 5. Gene and function of the “PON2 cluster of gene”** | |
| --- | --- |
| **Gene** | **Function** |
| **TRIM33** | Transcriptional corepressor^15^. Display potent antiviral activity against several viruses^16^. Acts as an E3 ubiquitin-protein ligase^17^. |
| **RAB40B** | Role in controlling invadopodia formation^18^. May be part of E3 ubiquitin ligase complex via the SOCS domain^19^. |
| **BIRC3** | Apoptotic suppressor^20^. Acts as an E3 ubiquitin-protein ligase regulating NF-kappa-B signaling^21^. |
| **RNF11** | Essential component of a ubiquitin-editing protein complex, comprising also TNFAIP3, ITCH and TAX1BP1, that ensures the transient nature of inflammatory signaling pathways^22,23^. |
| **MAP3K2** | Regulates the JNK and ERK5 pathways by phosphorylating and activating MAP2K5 and MAP2K7^24^. |
| **WDR36** | Involved in T cell activation and highly co-regulated with IL2^25^. |
| **LIN28B** | Acts as a suppressor of microRNA (miRNA) biogenesis^26-28^. |
| **WTAP** | Regulates G2/M cell-cycle transition by binding to the 3' UTR of CCNA2 (cyclinA2)^29^, which enhances its stability. Impairs WT1 DNA-binding ability and inhibits expression of WT1 target genes ^30^. May be involved in mRNA splicing regulation ^31^. |
| **SRPK2** | Phosphorylates RS domain-containing proteins, such as SFRS1 and SFRS2 on serine residues^32^. Role in spliceosome assembly and in mediating the trafficking of splicing factors^33^. |
| **RRM2B** | Plays a pivotal role in cell survival by repairing damaged DNA in a p53/TP53-dependent manner^34,35^. |
| **LRRC19** | A novel member of the leucine-rich repeat protein family, activates NF-kappaB and induces expression of proinflammatory cytokines. LRRC19 may play an important role in inducing innate immune responses in certain tissues such as the kidney^36^. |
| **CD93** | Receptor (or element of a larger receptor complex) for C1q, mannose-binding lectin (MBL2) and pulmonary surfactant protein A (SPA). May mediate the enhancement of phagocytosis in monocytes and macrophages upon interaction with soluble defense collagens. Now is thought to instead be involved in intercellular adhesion and in the clearance of apoptotic cells^37^. |
| **NEXN** | Involved in regulating cell migration through association with the actin cytoskeleton^38,39^. |
| **TTC22** | Protein with seven tetratricopeptide (TPR) repeats. TPR repeats are found in a variety of proteins and may mediate protein protein interactions and chaperone activity^40^. |
| **MGAT4A** | This gene encodes a key glycosyl transferase that regulates the formation of tri and multiantennary branching structures in the Golgi apparatus^41^. |
| **ROBO1** | The product of this gene is a member of the immunoglobulin gene superfamily and encodes an integral membrane protein that functions in axon guidance and neuronal precursor cell migration^42^. This receptor is activated by SLIT-family proteins, resulting in a repulsive effect on glioma cell guidance in the developing brain^43^. |
| **CPE** | This peripheral membrane protein cleaves C-terminal amino acid residues and is involved in the biosynthesis of peptide hormones and neurotransmitters, including insulin^44,45^. |
| **PHF12** | Acts as a transcriptional repressor. Involved in recruitment of functional SIN3A complexes to DNA. Represses transcription at least in part through the activity of an associated histone deacetylase (HDAC)^46,47^. May also repress transcription in a SIN3A-independent manner through recruitment of functional AES complexes to DNA. |
| **PEX12** | Required for protein import into peroxisomes^48,49^. |
| **KIAA0408** | Uncharacterized protein^50^ |
