# Supplementary material for: WTAP and BIRC3 are involved in the posttranscriptional mechanisms that impact on the expression and activity of the human lactonase PON2
Source: Cell Death Dis. 2020 May 7;11(5):324. doi: 10.1038/s41419-020-2504-2 (PMC7206036; doi:10.1038/s41419-020-2504-2)
Supplement: Supplementary file 22 — Table S6 [file 41419_2020_2504_MOESM22_ESM.docx]

| **Table 6. Alignment of the dodecameric conserved**  **sequences between the 21 genes included in the ”PON2 Cluster”** |
| --- |
| **1 32** |
| **RNF11 TGCACAAAAGTTTCCTTAAAATTCCTGGATGG**  **KIAA0408 TTTCTAATCTTTTCCTTAAAATTAATAGATAA**  **SRPK2 TGTTTATGCATTTCCTTAAAATTAATTGTAGA**  **PEX12 TGCCTATCCCTTTCCTTAAAATATAAGAAATA**  **TTC22 ATAACAACACTTTCCTTAAAATGTTGTCAACA**  **NEXN TTAATTTTTTTTTCCTTAAAATCACTTTTCTT**  **MGAT4A TTGTATATATTTTCCTTAAAATGTCTTCATTG**  **WDR36 TTATCTATATTTTCCTTAAAATATATCAATCT**  **MAP3K2 CTATATATTCTTTCCTTAAAATTCATGATTTT**  **TRIM33 AATAATTGTGTTTCCTTAAAATTACATGTTAG**  **BIRC3 CCTAATTTGGTTTCCTTAAAATTTTTATTTAT**  **CD93 CTCTGTCTCTTTTCCTTAAAATTGGGGGTAAG**  **CPE ATTAATCAACTTTCCTTAAAATAAATAGCCTC**  **LIN28B TCTCCAAACATTTCCTTAAAATAATCATGTAT**  **RRM2B AATACTCTCATTTCCTTAAAATAATTGTGATT**  **RAB40B ACTGCAACTTTTTCCTTAAAATAACTGCTTTT**  **WTAP AATGGAAATTTTTCCTTAAAATACAACACAAT**  **ROBO1 ACCTACAAGATTTCCTTAAAATCTCTAATAGA**  **LRRC19 GCACAATAAATTTCCTTAAAATTTCATAGCAA**  **PHF12 GCTTCAGTGATTTCCTTAAAATGACCTACTGA**  **PON2 GTGAATTATATTTCCTTAAAATGTGAGTGACC**  **Consensus ..........TTTCCTTAAAAT...t......** |
