# Supplementary material for: WTAP and BIRC3 are involved in the posttranscriptional mechanisms that impact on the expression and activity of the human lactonase PON2
Source: Cell Death Dis. 2020 May 7;11(5):324. doi: 10.1038/s41419-020-2504-2 (PMC7206036; doi:10.1038/s41419-020-2504-2)
Supplement: Supplementary file 23 — Table S7 [file 41419_2020_2504_MOESM23_ESM.docx]

| **Table 7. Primers and position of oligos on mRNA for quantitative real-time PCR** | | | | | | | | | |
| --- | --- | --- | --- | --- | --- | --- | --- | --- | --- |
|  | **Name** | **5’-3’** | **Position** | **Ref** | **Name** | **5’-3’** | **Position** | **Ref** | **Length** |
| **PON2 Iso 1** | 1-For | GACATACTTCCCAATGGTCTGGC | NM_000305.2  287-309 | p.a. | 1-Rev | TCTGGGTGGTTTACAACAAAGAGAT | NM_000305.2  525-501 | p.a. | 239 |
| **PON2 Iso 2** | 2-For | TCATAGACAACGAATTCAAGAATACA | NM_001018161.1  477-502 | p.a. | 2-Rev | CTGAATCAAATCCTTCTGCTAC | NM_001018161.1  749-728 | p.a. | 273 |
| **BIRC3** | 3-For | GACACATGCAGCCCGCTTTA | NM_182962.2  994-1013 | p.a. | 3-Rev | ACTGGCTTGAACTTGACGGATG | NM_182962.2  1246-1225 | p.a. | 253 |
| **WDR36** | 4-For | CTGGGACTCGCCTTGGATG | NM_139281.2  1900-1918 | p.a. | 4-Rev | TTGAGAGGAGCCGAGTCCAAC | NM_139281.2  2120-2100 | p.a. | 221 |
| **TRIM33 iso A** | 5-For | GAGCCTGTTCCTGCTTCGATAC | NM_015906.3  3034-3055 | p.a. | 5-Rev | TGTGTGTCTGCATAAACTTGAACAAC | NM_015906.3  3239-3214 | p.a. | 206 |
| **TRIM33 iso B** | 6-For | GAGCCTGTTCCTGCTTCGATAC | NM_033020.2  3034-3055 | p.a. | 6-Rev | GCTACTTCTGAATCAGCTTCATTAAACC | NM_033020.2  3221-3194 | p.a. | 188 |
| **RAB40B** | 7-For | CCGGGCCTACGACTTTCTG | NM_006822.2  150-168 | p.a. | 7-Rev | CCTGGCCTGAAGTATCCCAGA | NM_006822.2  346-326 | p.a. | 197 |
| **RNF11** | 8-For | GATGGGGAACTGCCTCAAATC | NM_014372.4  484-504 | p.a. | 8-Rev | CTGGCTAGGTGTTGGGTGGTAG | NM_014372.4  649-628 | p.a. | 166 |
| **WTAP** | 9-For | TAATGGCGAAGTGTCGAATGC | NM_001270531.1  682-702 | p.a. | 9-Rev | CTGCATACCCTCTACTTCTTCATCAA | NM_001270531.1  869-844 | p.a. | 188 |
| **WTAP KIAA** | 10-For | GTAATGCGACTAGCAACCAAGG | NM_152857.2  441-462 | p.a. | 10-Rev | TGAGTCTTGGTGTGGAAACGAGT | NM_152857.2  763-741 | p.a. | 323 |
| **ACTB** | 11-For | CATCATGAAGTGTGACGTGGACA | NM_001101.4  1035-1057 | p.a. | 11-Rev | CCTAGAAGCATTTGCGGTGGA | NM_001101.4  1321-1301 | 51 | 287 |
| **GAPDH** | 12-For | CGGATTTGGTCGTATTGGGC | NM_002046.6  103-122 | 52 | 12-Rev | TCGCTCCTGGAAGATGGTGAT | NM_002046.6  316-296 | 52 | 214 |

p.a.=**this work**
