# Supplementary material for: WTAP and BIRC3 are involved in the posttranscriptional mechanisms that impact on the expression and activity of the human lactonase PON2
Source: Cell Death Dis. 2020 May 7;11(5):324. doi: 10.1038/s41419-020-2504-2 (PMC7206036; doi:10.1038/s41419-020-2504-2)
Supplement: Supplementary file 24 — Supplementary Figure Legends [file 41419_2020_2504_MOESM24_ESM.docx]

**Legends to figures**

**Fig. S1. Scheme of the PON2 gene and of the seven mRNA isoforms (and our RefSeq codes).**

**a** The boxes indicated the exons; the arrows and capital letters indicated the oligonucleotides used for the PCR. The Isoforms 5 and 6, that should give rise to very short protein of 50 and 84 aa, were identified, but as very faint bands. **b** Alignment of Iso 1 canonical and PP sequences.

**Fig. S2. Control of immunoprecipitation**

In **b** is shown the result of anti-Ubiquitin IP as obtained in **a** but omitting the antibody. No IP band was observed in this case.

**Fig. S3. Time course of 3OC12HSL treatment up to 40 minutes and detection by western blot with anti-PON2 antibodies.**

**Fig. S4.** Spectrum and fragmentation table for the identified peptide showing ubiquitination at position 29 in the canonical isoform.

**Fig. S5.** Spectrum and fragmentation table for the identified peptide showing the N-ter of PP Iso1.

**Fig. S6.** Spectrum and fragmentation table for the identified peptide showing ubiquitination at position 159.

**Fig. S7.** Spectrum and fragmentation table for the identified peptide showing N-ter of canonical not modified Iso 1.

**Fig. S8.** Spectrum and fragmentation table for the identified peptide showing ubiquitination at position 29 in the PP Iso 1.

**Fig. S9.** Spectrum and fragmentation table for the peptide demonstrating the existence of Iso 2.

**Fig. S10.** Spectrum and fragmentation table for the identified peptide showing ADP ribosylation at position 124.

**Fig. S11. SAXS analysis**

SAXS patterns obtained from PON2 (**a**) and 123-134delrPON2 (**b**). Data represent the average intensity, I(q), as a function of the momentum transfer, q, of two independent experiments, respectively. The black solid lines represent the reciprocal space fit of scattering computed for the final P(r) function to data. The red continuous line is the scattering calculated from the EOM results. The inset of each spectrum is the Guinier plot and the solid lines represent the extrapolation of data to *q* = 0, for *qR_g_* < 1.3.

The corresponding distance distribution functions, *P*(*r*), for PON 2 and 123-134delrPON2 are represented in **c** and **d**, respectively. The Kratky plot (*q*^2^*I*(*q*) vs. *q*) for PON2 and 123-134delrPON2 are represented in **e** and **f**, respectively.

**Fig. S12. EOM study**

Results of the EOM study showing the distribution of *R_g_* (**a**) and *D_max_* (**b**). The distributions of the random pool are shown in black, and those of the selected ensemble for 123-134delrPON2 are in red.

The experimental X-ray scattering data for 134delrPON2 are shown as circles **(c)**. The continuous red line is the scattering curve calculated from the best optimization obtained from the EOM analysis.

**Fig. S13.**  **EOM ensemble yielding a set of 6 models**

**a** The backbone representation of the six conformation of the 134delrPON2 selected by the EOM analysis.

**b** The DAMMIN reconstruction is shown for comparison.

**c** All the structures shown in **a**, are superimposed and fitted into the SAXS based *ab initio* reconstruction of the 134delrPON

**Fig. S14. Analysis of expression and optimization of siRNA experiments for WTAP and WDR36**

**(a)** Basal expression of four E3 Ubiquitin ligases (BIRC3, RNF11, RAB40B; TRIM33 Iso A and B) two RNA binding proteins (WTAP and WTAP KIAA; WDR36) PON2 and ACTB and GAPDH as controls.

Dose response curves with 12.5, 25 and 50 nM of silencing oligos for WTAP (**b**) and WDR36 (**c**). GAPDH was used as control.

**Fig. S15. Pull down of WTAP related proteins with a specific RNA Binding Sequence**

**(a)** Scheme of the experiment of pull down from HeLa extract of WTAP protein able to bind the conserved sequence of the "cluster of genes". **(b)** Western blot with anti-WTAP antibody of proteins that specifically bind the biotinilated (and phosphorotioate) oligo.
